# Leveraging Consensus Docking Approaches for Human Mitochondrial Complexes I and III

**DOI:** 10.1021/acs.chemrestox.5c00348

**Published:** 2025-12-30

**Authors:** Karin Grillberger, Viktoria Magel, Marcel Leist, Gerhard F. Ecker

**Affiliations:** † Department of Pharmaceutical Sciences, 27258 University of Vienna, Josef-Holaubek-Platz 2, Vienna 1090, Austria; ‡ In vitro Toxicology and Biomedicine, Department Inaugurated by the Doerenkamp-Zbinden Foundation, 26567 University of Konstanz, Universitätsstraße 10, Konstanz 78457, Germany

## Abstract

Although recent progress has been made, structure-based methods such as molecular docking are still underexplored in the context of toxicity prediction. These approaches offer added value, particularly in addressing challenges such as activity cliffs—i.e., caused by stereoisomerism—that are difficult to capture by conventional Quantitative Structure–Activity Relationship (QSAR) methods. In this study, we investigated the ability of docking scoring functions and protein–ligand interaction fingerprints to rank the potential hazard of compounds targeting the human mitochondrial complexes I and III (CI, NADH:ubiquinone oxidoreductase and CIII, cytochrome bc_1_ complex). We applied an induced fit docking protocol to account for binding site flexibility and performed a set of binding energy minimizations for rescoring of representative binding modes. Both individual scoring functions and consensus scoring approaches achieved acceptable rank correlation to experimentally derived data from CIII (Spearman *r*: 0.89 and 0.86). Moreover, consensus interaction fingerprints that combine molecular interactions from both docking outputs captured differences of inhibitor subtypes at CIII. Follow-up in vitro testing confirmed an isomerism-dependent activity cliff of E-/Z-Fenpyroximate at CI. These findings support the utility of using consensus docking and scoring as a screening-level tool for prioritizing compounds based on interpretable predicted relative binding affinities at CI and CIII.

## Graphical abstract

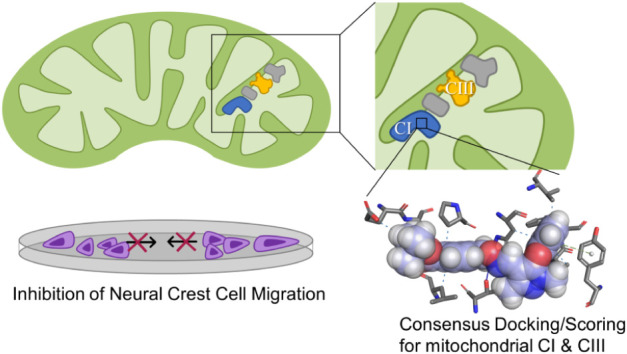

## 1 Introduction

Toxicity testing in the 21st century is anticipated to envision a shift from traditional in vivo testing toward the implementation of new approach methods using in vitro and in silico methods.^1,2^ This transition is also reflected by regulatory instances, for example, in the acceptance of quantitative structure–activity relationship (QSAR) for mutagenicity assessment of impurities of pharmaceuticals,^3^ or the establishment of the OECD QSAR toolbox.^4–6^

One of the toxic end points that still causes drug developments to fail is mitochondrial toxicity,^7,8^ although several machine learning models for prediction of mitochondrial toxicity^9–13^ or inhibition of the electron transport chain^14,15^ have been published. In this regard, it is worth mentioning that common data standardization processes in machine learning-based predictions for toxicity end points using chemical structures imply the removal of stereochemistry and geometrical isomerism.^10,16^ This is, for example, caused by the inability of many commonly used chemical descriptors to efficiently encode chirality or because of missing stereochemistry information.^16^ Nevertheless, in a biological context, it is important to consider chirality and isomerism of molecules.^17–19^ Stereochemical features can drive differences in on- and off-target interaction, hence also in toxicity outcomes due to different pharmaco- and toxicodynamic profiles.^20^

Taken together, consideration of stereochemistry, geometric isomerism, tautomerism, and protonation states are important factors that determine the quality of in silico modeling outcomes. Molecular docking is a technique that can consider those factors, thus adding an important value and complementarity for toxicity prediction methods. There have also been endeavors to use molecular docking for toxicological end points^21–23^ and to combine its output with in vitro experiments related to mitochondrial dysfunction.^24,25^

Besides interactions of chemical stressors with the mitochondrial membrane, or mitochondrial DNA (mtDNA), disruption of the electron transport chain (ETC) is one of the key mechanisms leading to mitochondrial toxicity.^8^ The ETC is responsible for oxidative phosphorylation and is composed of four mitochondrial complexes (CI–IV) that can be organized as circular megacomplexes in the inner mitochondrial membrane. CI, III, and IV are also involved in pumping protons into the intermembrane space. Thus, they are building up the proton gradient, which is necessary for ATP synthesis, which in turn is essential for important physiological functions of cells.^26^

Therefore, disruption of the function of the ETC leads to mitochondrial dysfunction and is considered a key event for adverse outcome pathways linked to toxicity end points. For example, inhibition of mitochondrial complex I (CI, NADH:ubiquinone oxidoreductase) has been linked to neurodegenerative effects like Parkinsonian motor deficits.^27,28^ Additionally, inhibition of mitochondrial complex III (cytochrome bc_1_ complex) was recently proposed as a molecular initiating event, potentially triggering neurodevelopmental defects.^29^ Furthermore, CI and CIII have also been suspected to play an important role in cancer, inflammation, and other diseases.^30,31^

The aforementioned points highlight the importance of in-depth evaluation of the structural basis of CI- and CIII-inhibition and their chemical stressors. Herein, we applied a consensus docking protocol in a cross-docking mode to balance out potential limitations of individual scoring functions. We applied induced fit docking using two classical state-of-the-art docking algorithms, that employ different ligand placement strategies (exhaustive search and genetic algorithm for Glide^32^ and GOLD,^33^ respectively) in order to improve the reliability of predicted binding modes and protein–ligand interactions derived thereof. The increasing use of consensus approaches to predict the potential toxicity of chemicals is also reflected in recent endeavors of consensus QSAR approaches such as CERAPP,^34^ COMPARA,^35^ or others.^36–38^ For this study, a set of 28 compounds was selected (see Section 2.1, Table S1), comprising commercially used fungicides, acaricides, and some drug candidates, that display selectivity toward either of the two mitochondrial complexes of interest (CI and CIII), or represent mitochondrial uncouplers (protonophores that facilitate proton movements across the membrane, without directly targeting the mitochondrial complexes). Regarding the protein binding sites, we focused our analysis on the primary inhibitor binding sites of CI and CIII. For CI, we selected the deep quinone/quinol binding site (Qd) located between the 49-kDa (NDUFS2) and PSST (NDUSF7) subunits, as this site has been shown to accommodate classical CI-inhibitors like rotenone^39,40^ and piericidin A.^41,42^ In the case of CIII, we docked into the quinol oxidation site (Qo) given that the fungicides included in this study have been previously reported to bind selectively to this site of CIII.^43–45^ Moreover, we highlight the importance of consideration of cis/trans isomerism, which was validated by specific in vitro assays.

## 2 Materials and Methods

### 2.1 Protein and Ligand Preparation

Protein structures of human mitochondrial complexes I and III (CI and CIII, PDB-IDs 5xtd and 5xte)^26^ were retrieved from the Protein Data Bank.^46^ Proteins and ligands were prepared at pH 7.4 ± 0.5 using Protein Preparation Workflow^47^ and LigPrep,^48,49^ while keeping all other options as default. For preparation of proteins and ligands, the OPLS4 force field was used.^50^ The grid centers for CI and CIII were defined by structural alignment with cocrystallized bovine structures (PDB-IDs 7vbz^40^ and 1sqq,^44^ respectively), where the centroid of amino acids lining within 5Å of the ligand was used. This corresponds to the coordinates *X*: 222.61, *Y*: 167.24, *Z*: 281.49 for CI, and *X*: 226.08, *Y*: 319.31, *Z*: 232.34 for CIII. Only protein chains relevant for the binding sites of interest were retained. For CIII, we kept chain V, and chains B, C, E, P, N, Q, j, and s were retained for CI.

In order to adapt the binding site conformation of 5xte (CIII) to be more similar to bovine cocrystallized complexes, we changed the original rotamer of Glu271 using the build tool of Maestro (Figure S1), hence reducing the initial steric hindrance, which was not resolved by the steps described hereafter. Since more conformational changes of amino acid side chains in the binding site were expected upon ligand binding, we proceeded to apply induced fit docking (IFD) protocols.

Regarding the ligands, we extended the previously selected set of compounds,^29^ by enriching it with those substances from literature where information about CI-/CIII-specificity was available, in order to have a more chemically diverse selection. Table S1 also lists the references reporting the major mode of action, based on functional or binding assays on the electron transport chain. In this study we specifically excluded CIII-Qi-site inhibitors, hence Qo-site inhibitors are termed as CIII-inhibitors in the following sections. Since previous crystallographic and cryo-EM studies suggest that the majority of CI-inhibitors target the deep quinone (Qd) binding site of CI,^39,40,42,51^ we focused in this study only on the Qd site, neglecting potential different binding sites of CI.

### 2.2 Induced Fit Docking (IFD) Protocols

The first IFD protocol that has been applied in this study was within the Schrödinger software suite version 22–4,^52–54^ applying the standard sampling procedure that produced up to 20 poses per ligand. Here, the Prime module is used to sample residue side chain conformations and for ligand binding site minimization.^52,55^ It also includes a redocking step into the optimized binding site.^54^ The IFD output was subjected to an interaction-fingerprint-based clustering module within Schrödinger, and the highest populated cluster was analyzed for further pose selection. This cluster was also used to generate an interaction fingerprint, where the most frequent interactions informed the second docking protocol, GOLD with flexible side chains.^33^ The IFD protocol of the Schrödinger software suite included residues lining within 5 Å of the ligand for the refinement step, hence the flexible residues depend on the respective ligand poses, whereas in GOLD a fixed list of flexible side chains has to be provided in the configuration of the docking protocol. GOLD version 2020.2.0 was used, keeping the prepared input structures of proteins and ligands the same as for the first IFD protocol. Up to 100 poses per ligand were generated, and early termination was switched off. Following binding site side chains were set to be flexible for CI and CIII, respectively: Met94C, His92Q, Tyr141Q, Met185Q, Thr189Q, Asp193Q, Phe200Q and Phe128 V, Tyr131 V, Met138 V, Ile146 V, Glu271 V, Tyr273 V, Phe274 V, Tyr278 V, and Leu281 V. The ChemPLP score was selected as the default scoring function, and rescoring of the generated docking poses was done with GoldScore and ChemScore.

For pose selection, we first applied interaction fingerprint clustering to the docking poses within Maestro 22–4, and analyzed the highest populated cluster. If a ligand had no poses in that cluster we checked for poses in the next highest populated cluster, to obtain one representative binding mode per ligand. Poses were selected within the clusters according to docking score and visual inspection. Visual inspection was carefully conducted to pick the most appropriate binding modes when cocrystallized structures from other organisms were available (see Table S1). For the docking output generated from GOLD, the best-scoring poses according to the ChemPLP Fitness score were taken for ligands where no binding information was available. When cocrystallized structures from other species were available, we visually inspected the top 3 poses, and in special cases, all generated poses, to select a binding mode consistent with the available structural information and to ensure accurate interpretation of binding modes and interactions.

### 2.3 Binding Energy Calculations

Representative binding poses from both docking programs were subjected to binding energy calculations, using the VSGB 2.1 implicit solvation model^56^ and OPLS4 force field.^50^ Since GOLD applied different atom parametrizations, the extracted protein–ligand complexes needed to be preprocessed with the Protein Preparation Workflow^47^ before subjection to relative binding energy calculations.^47^ We applied the Embrace Minimization tool utilizing MacroModel,^57,58^ keeping the ligand and residues within 5 Å as flexible substructures, while freezing the rest of the protein structure. Two modes of binding energy calculations came from MacroModel, the Desolvation Energy and Interaction Energy (MBAE Del Total Energy and MBAE ASET Total Energy). Similarly, binding energy calculations using Prime MM-GBSA (MM-GBSA dG Bind) were based on the ligand and included residues lining within 5 Å.

### 2.4 Consensus Scoring Approaches

In total, 11 different scoring functions and binding energy calculations were used to derive consensus scores. This aims to balance out individual limitations of scores. To avoid incompatibilities of docking scores due to different scales and dependence on protein size, relative rankings were derived from the scoring functions. Ranking directions of individual scoring functions were considered, i.e., ascending for docking score, IFDScore, and binding energy calculations, and descending for fitness functions from Gold (negative sum of the component energy terms). Thus, each ligand received a relative rank across the scoring functions, where low rank numbers indicate favorable binding. The resulting ranks from individual scoring functions were combined in a consensus ranking using two approaches, aggregated ranking and exponential consensus ranking.^59^

The aggregated rank approach, sometimes referred to as rank by rank, is a commonly used consensus scoring technique that averages the ranks of individual scoring functions. Relative rank numbers are added for all ligands, which results in a Borda Count. Ranking the Borda Count in ascending order yields the aggregated rank.

The exponential consensus rank (ECR) approach assumes an exponential distribution for each rank from the scoring functions that are combined.^59^ The σ-parameter was constantly kept at 10, since it has been shown that ECR is nearly independent of it. High ECR scores indicate favorable binding.^59^

### 2.5 Consensus Interaction Fingerprints

To generate interaction-based fingerprints, MM-GBSA-minimized poses from Maestro and GOLD were exported as PDB files and analyzed with PLIP.^60^ The generated XML files of PLIP are used to extract the information about the protein–ligand interactions. This processing step results in a table that lists every interaction type, residue number, residue type, protein chain of the residue (important if multiple protein chains compose the binding site which is the case i.e., for CI), and the name of the ligand the interaction is directed to. Protein–ligand interactions that were present in both binding modes were combined by merging on the levels of interaction type, residue number, residue type, protein chain of the residue, and ligand name. Subsequently, the table was transformed into a binary fingerprint that encodes the presence or absence of a combination of interaction type, amino acid, amino acid number and protein chain, i.e., interaction_type@number_of_amino acid@amino acid@chain. The interaction patterns were visualized using matplotlib^61^ and seaborn^62^ libraries, and data processing in Python was done with pandas,^63^ numpy,^64^ scipy,^65^ and scikit-learn.^66^ For comparison of the consensus interaction fingerprints, we also generated chemical substructure-based fingerprints in terms of MACCS keys^67^ (Figure S5C). Visualizations of representative binding modes were generated in PyMOL version 2.5.^68^

### 2.6 Circular Migration of Neural Crest Cells (cMINC) Assay

Neural crest cells (NCC) were differentiated, as described earlier, from the human induced pluripotent stem cell (hiPSC) line IMR90_clone_#4 (WiCell, Madison, WI, USA), following the modified protocol of Mica et al.^69^ and according to Nyffeler et al.^70^ and Dolde et al.^71^

To perform the cMINC assay, NCCs were thawed in N2–S medium and seeded on the day of migration-1 (DoM-1) on a 96-well polystyrene plate (Corning, Glendale, AZ, USA) around stoppers, to create a cell-free area of 2 mm diameter (Platypus Technologies, Madison, WI, USA). The cells were resuspended in N2–S medium, supplemented with 20 μg/mL of the cytokines EGF and FGF, and then counted and seeded around the stoppers at a density of 95,000 cells/cm^2^. After 24 h, the stoppers were removed, and the medium was exchanged. The removal of the stoppers initiates migration of the NCCs into the cell-free area. For the treatment, the 5x-concentrated toxicant solution (25 μL) was added to 100 μL medium at 24 h after stopper removal.

On day 2, cells were stained with final concentrations of 533 nM calcein-AM and 1 μg/mL H-33342 (both from Sigma, Steinheim, Germany) and observed using two different channels on the Cellomics ArrayScan VTI imaging microscope (Thermo Fisher, Pittsburgh, PA, USA) to assess viability and migration. For the migration, four pictures were taken in the region of interest (ROI) (5× magnification objective). Migration data were obtained using the software “Ringassay” (http://invitrotox.uni-konstanz.de/), which can estimate the previously cell-free area and count the number of H-33342 and calcein-AM double-positive cells. Cell viability was assessed by taking four pictures outside of the ROI with a 10× magnification objective. Viable cells were defined as H-33342 and calcein-AM double-positive cells and calculated by an automatic algorithm of the ArrayScan VTI 700 Series software as described earlier.^70,72^ A run was rejected if treatment with the positive control, 200 nM cytochalasin D (Sigma, Steinheim, Germany), did not inhibit migration by at least 25% and cell viability was below 90%. Renormalization of the concentration–response curves was performed as previously described.^73^ The calculations of the benchmark concentrations 10 for viability (BMC_10_ (V)) and 25 for migration (BMC_25_ (M)) were performed with the online available BMC software (http://invitrotox.uni-konstanz.de/).^74^

### 2.7 Oxygen Consumption Test to Assess Complex I Inhibition

To investigate the inhibition of complex I (CI) after Fenpyroximate exposure, NCCs were permeabilized and complex-specific substrates and inhibitors of CI were injected as described earlier.^75^ In detail, cells were permeabilized with MAS buffer (220 mM mannitol, 1 mM ADP, 70 mM sucrose, 10 mM KH2PO4, 5 mM MgCl2, 2 mM HEPES, 1 mM EGTA, 4 mg/mL fatty acid-free BSA, pH = 7.2) supplemented with 25 μg/mL digitonin. Seahorse measurements were started directly afterward, and basal oxygen consumption rates (OCR) were assessed. Fenpyroximate isomers were loaded into the injection port at a 10-fold higher concentration. Upon injection of 56 μL into 500 μL assay medium, the final in-well concentration was 5 μM. Then, CI activity was assessed. For this, CII was inhibited (5 mM malonate) and substrates for CI (2 mM l-glutamine, 2.5 mM malic acid, 5 mM pyruvic acid) were added. Data were compared to the solvent control. Details and assay validation have been provided earlier.^75,76^

### 2.8 Compound Handling

Z-Fenpyroximate (Fisher Scientific, Hampton, NH, USA) and E-Fenpyroximate (Biomol, Hamburg, Germany) were both prepared as 50 mM stock solutions. The compounds were diluted in dimethyl sulfoxide (DMSO) and stored as aliquots of 10 μL at −20 °C.

## 3 Results

Herein, we applied a consensus docking protocol in a cross-docking set up to balance out potential limitations of individual scoring functions and to evaluate target selectivity of this approach. We implemented induced fit docking by employing two classical state-of-the-art docking algorithms, aiming to enhance accuracy of predicted binding modes and protein–ligand interactions that were derived thereof. A cross-docking setting was employed, where specific inhibitors of respiratory chain complex I were docked in complex III, and vice versa. Hence, for this study, a set of 28 compounds was selected (see Section 2.1), comprising commercially used fungicides, acaricides, rodenticides, and some drug candidates, that display either selectivity toward one of the two mitochondrial complexes of interest, or lead to mitochondrial uncoupling without direct interaction with CI or CIII (Table S1). Regarding the protein binding sites, the focus was on the main inhibitor binding sites, the quinone/quinol binding site of CI (Qd), which is located between the 49-kDa (NDUFS2) and PSST (NDUSF7) subunits, and the quinol oxidation site (Qo) that is close to the low potential heme (heme b_L_) of CIII. Moreover, we emphasize the importance of consideration of geometrical E-/Z-isomerism, as exemplified by distinct effects of E-Fenpyroximate on CI, which were further confirmed by subsequent specific in vitro assays.

### 3.1 Scoring-Based Classification

First, we checked if individual scoring functions would be able to differentiate between preferable binders and nonbinders. For this docking-based classification task, we plotted kernel density estimate (KDE) plots of the ranks of each scoring function (Figure S2, Figure S3). Interestingly, the uncoupling compounds (chlorfenapyr, cyazofamid, and fluazinam) received low ranks in both mitochondrial complexes, indicating non- or less favorable binding at CI and CIII. In the case of CI (Figure S2), the probability density curves of CI- and CIII-inhibitors and uncoupling compounds were separated best using the scoring from the IFDScore. Hence, the intersection point of the curves can be used as a criterion for a scoring-based classification (Table S2).^21,77^ Accordingly, ligands having an IFDScore lower than −3170.75 were classified as CI-inhibitors, less negative scores labeled the ligands as non-CI-inhibitors (Figure 1A). A confusion matrix (Figure 1B) and classification report based on the predicted and actual classifications revealed an acceptable balanced accuracy (BAC) of 0.786 and a Matthews Correlation Coefficient (MCC) of 0.577 for this scoring-based CI-inhibitor classification task. Interestingly, stigmatellin was classified as CI-inhibitor by the IFD threshold, although it is known to inhibit CIII in a nanomolar concentration range, whereas effects on CI are reported only for micromolar concentrations.^78,79^ It has to be noted that this approach worked best only in the case of CI and the IFDScore. Thus, this proposed threshold should be used with caution as a classification criterion, also because of the limited sample size of this cross-docking classification study (14 CI-inhibitors, 14 non-CI-inhibitors (11 CIII-inhibitors and 3 uncouplers)).

**Figure 1:**
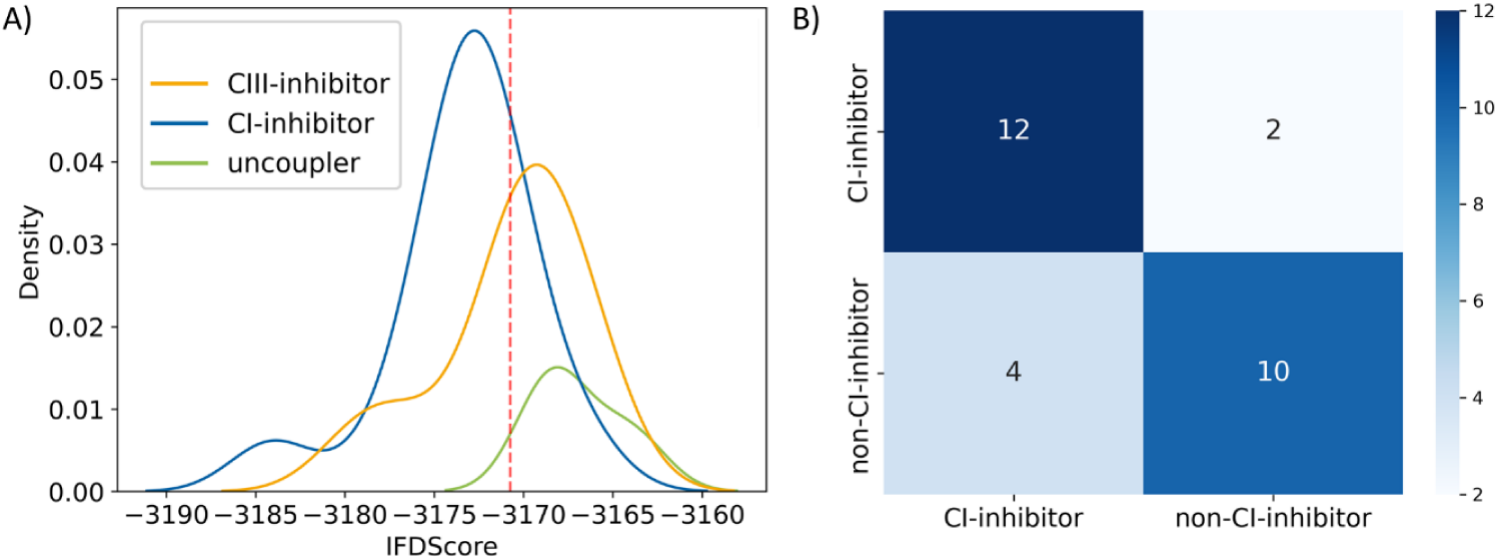
Kernel density estimate (KDE) plot (A) and confusion matrix (B) derived from IFDScore-derived classification of 14 CI- and 14 non-CI-inhibitors (CIII-inhibitors and uncouplers) from docking into human CI. (A) The relative densities of CI- and CIII-inhibitors and uncouplers are colored in blue, yellow, and green, respectively, and are plotted against their respective IFDScores from docking into human CI. The intersection of the KDE curves between the CI- and non-CI-inhibitors is marked as a red dashed line at the IFDScore of 3170.75. (B) The confusion matrix was computed with the scikit learn library in Python.^66^

To determine if the observed performance of the IFDScore generalizes, we next examined the individual scoring functions and binding energy calculations for a scoring-based classification in the context of mitochondrial CIII. In contrast to CI, at CIII, scoring functions that evaluated binding poses generated by GOLD revealed the best binder versus nonbinder classification performance (Figure S3, Table S2). At CIII, the data set was a bit more imbalanced (11 CIII-inhibitors and 17 non-CIII-inhibitors; notably, deguelin could not be docked in CIII with the IFD protocol of Maestro), so we continued to evaluate with MCC and BAC. The GoldScore Fitness function determined the intersection point at a value of 46.20, where higher scores were labeled as CIII-inhibitors (Figure S4A). This resulted in an MCC of 0.781 and a BAC of 0.896. The ChemPLP Fitness function of GOLD reported an intersection point at a score of 80.52, which corresponds to an MCC of 0.647 and a BAC of 0.823 (Figure S4B). Also, docking poses from GOLD that were subjected to MM-GBSA binding energy calculations (MMGBSA dG Bind (GOLD)) achieved acceptable classification performance (MCC = 0.602, BAC = 0.807) when CIII-inhibitors were classified by MMGBSA dG Bind values lower than −59.30 (Figure S4C).

### 3.2 Consensus Scoring Results

Subsequently, we continued to evaluate if a combination of scoring functions would lead to increased robustness in the selection of top-ranking compounds. It has been found previously that a consensus scoring approach enhances success rates in virtual screening campaigns.^59,80^ Therefore, we evaluated this approach in a toxicologically relevant context.

Considering the different scales, units, and ranking directions of the scoring functions that are used in this study (see Section 2.4), relative rankings and consensus ranks are used for further comparison. Otherwise, it would be difficult to combine the results from the two docking programs. Moreover, we observed that the rankings of different scoring functions are not necessarily correlated within and across the targets, CI and CIII.

From the split triangle correlation heatmap (Figure 2), it becomes evident that scoring functions exhibit different correlation patterns within and across the two protein structures, as we suspected based on the differences in performance of scoring-based classification tasks. If the individual scoring functions would correlate well among each other independently of the protein target, the upper and lower triangles of the heatmap would be mirrored. However, as shown in Figure 2, this is not the case. For instance, MM-GBSA dG Bind-derived rankings did not correlate well with other scoring functions in CI, while in CIII, they align more closely. In contrast, the consensus scoring approaches ECR and aggregated ranking demonstrate consistent performance in both protein targets. Also, as expected, the ECR and aggregated ranking approaches are highly correlated with each other (Spearman *r* = 0.93 and 0.95 for CI and CIII, respectively). Both consensus approaches combine the rankings of multiple scoring functions using relative rankings. ECR was developed to take the best molecules from either program by essentially rewarding consistently highly ranked compounds,^59^ whereas the aggregation approach averages the relative ranks. These findings support the hypothesis that the performance of individual scoring functions is dependent on protein structures, whereas consensus approaches might overcome these target-dependent offsets.

**Figure 2:**
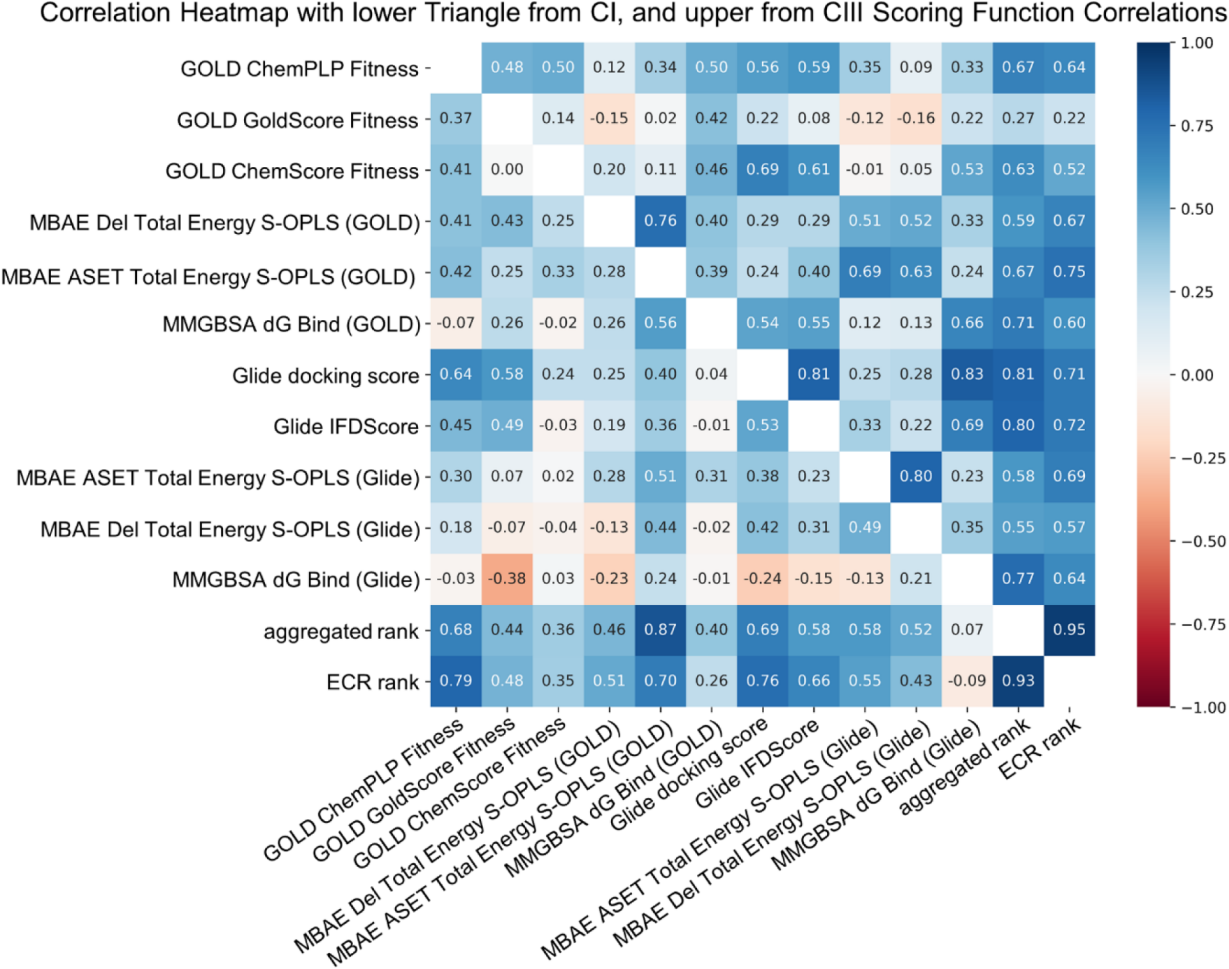
Correlation heatmap of relative rankings from individual scoring functions and consensus ranks. The lower and upper triangles correspond to the docking output of CI and CIII, respectively. The heatmap is colored according to the Spearman coefficient: blue indicates positive and red indicates negative rank correlation; white and light shades of blue and red indicate no or little rank correlation of the scoring functions and consensus ranks.

#### 3.2.1 Combination of Consensus Scoring and In Vitro Assay Results at CI

One main advantage of docking studies is the consideration of the stereochemistry and isomerism of chemicals in a chiral environment (protein binding site). This is often disregarded in models that are based on chemical structure alone because stereochemistry removal is a common step in data standardization pipelines. Moreover, most of the commonly used descriptors based on chemical structures cannot efficiently encode stereochemistry.^16^ Among the compounds that were analyzed, the acaricide Fenpyroximate exhibits E-/Z-isomerism. From most activity reports, it does not become evident, which isomer is determining the activity. Also, metabolism-induced isomerization was mentioned in an FAO/WHO report,^81^ however, this would probably require a specific enzyme or extreme pH changes, considering that the isomeric double bond is within the oxime group of the molecule. To address this uncertainty, we evaluated both the scoring output and binding poses to determine whether one isomer showed a preferable binding to CI.

Hence, we analyzed E- and Z-Fenpyroximate (E- and Z-FPM) in more detail regarding the individual scores and consensus scoring results. When evaluating the isomeric pair, the trend of E-FPM having overall better scoring compared to the Z-isomer became evident from consensus scoring already: ECR values were higher for E-FPM than for Z-FPM, when calculated from both, the whole ligand data set (4.28 and 2.90) or from the CI-inhibitor subset (6.48 and 5.27). Accordingly, also the aggregated ranking reported Z-FPM at worsened ranks. For in-depth analysis and for direct comparison of E- to Z-FPM, we also evaluated the actual scores of the scoring functions that compose the consensus scores (ECR and aggregated rank). Nine out of 11 scoring functions reported that E-FPM had more favorable energetic contributions upon binding compared to the Z-isomer (Table 1). Also, the IFDScore of Z-Fenpyroximate (Z-FPM) was very close to the scoring-based CI-inhibitor classification threshold (IFDScore < −3170.75) which has been defined by KDE plots (Figure 1). Although the IFDScore would be sufficient for differentiation at CI, this is not the case in CIII, hence target dependence of scoring functions is not compensated by the IFDScore. Taken the consensus and individual scoring results together, it indicated less favorable binding of Z-FPM at CI compared to other selective CI-inhibitors.

**Table 1:** Docking Scores of E- and Z-Fenpyroximate at CI^a^

Scoring Function	E-Fenpyroximate	Z-Fenpyroximate
GOLD ChemPLP Fitness*	86.32	80.59
GOLD ChemScore Fitness*	28.77	27.47
GOLD GoldScore Fitness*	62.01	51.33
MMGBSA dG Bind (GOLD)	–53.22	–40.61
MBAE ASET Total Energy-S-OPLS (GOLD)	–251.30	–244.66
MBAE Del Total Energy-S-OPLS (GOLD)	–162.35	–168.63
Glide docking score	–9.14	–8.70
Glide IFDScore	–3173.87	–3170.89
MMGBSA dG Bind (Glide)	–56.31	–34.79
MBAE ASET Total Energy-S-OPLS (Glide)	–241.70	–235.88
MBAE Del Total Energy-S-OPLS (Glide)	–203.43	–206.22

a Scoring functions marked by an asterisk use a genetic algorithm for ligand placement and higher scores (i.e., more positive values) to indicate better predicted affinity, which is unlike the remaining scoring functions.

In addition to the scoring-based divergence of this isomeric pair, we also qualitatively investigated specific protein–ligand interactions. According to a photoaffinity labeling study that was conducted in a bovine complex I, FPM labeled the PSST (chain C in 5xtd) and the 49 kDa subunit (chain Q in 5xtd), with photoreactive groups close to the pyrazole and ester group, respectively.^82^ The proposed binding modes from docking also show a similar orientation of E-FPM (Figure 3A). The pyrazole group forms hydrogen bonds to Gly85 and Thr83 of the PSST subunit and to Ser205 in the ND1 subunit (chain s). Additionally, several hydrophobic interactions are formed in the 49 kDa subunit to side chains of Pro89, Thr189, and Leu192, as well as to Met93 and Phe110 of the PSST chain (Figure 3A).

**Figure 3:**
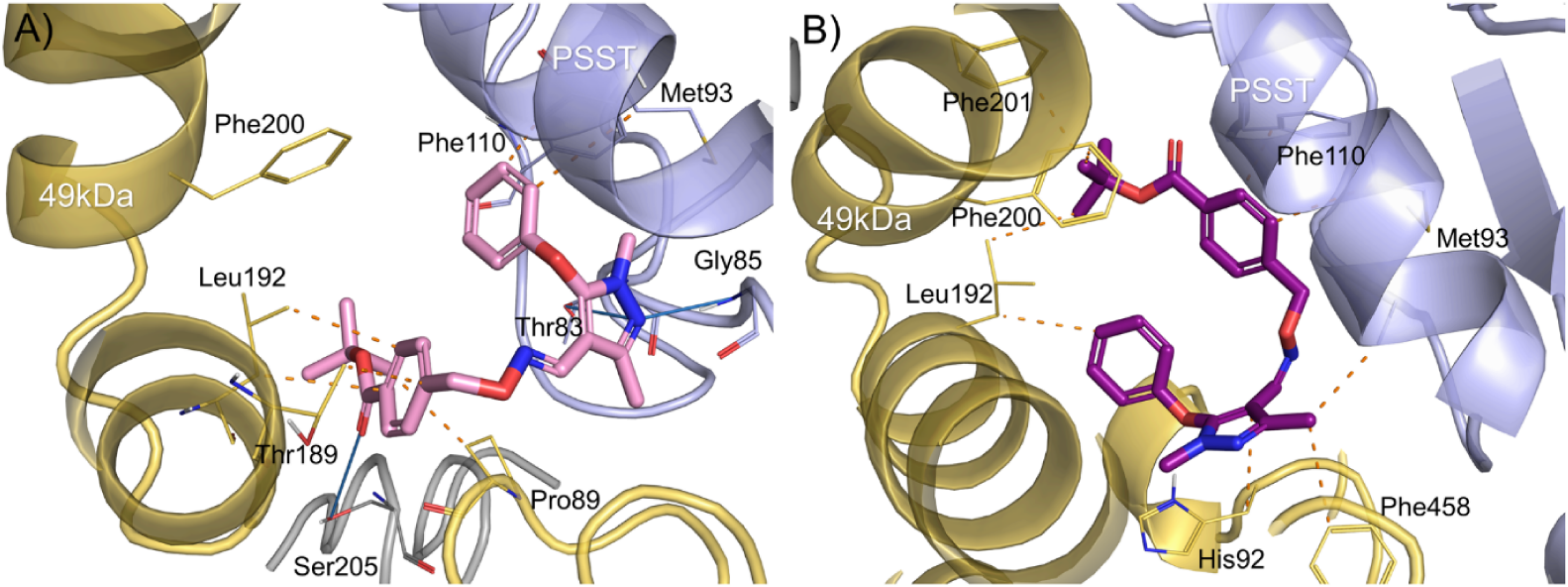
Proposed binding modes of (A) E-Fenpyroximate (E-FPM) and (B) Z-Fenpyroximate (Z-FPM) in the Qd binding site of human CI. Hydrophobic interactions are indicated by orange dashes, and hydrogen bonds are depicted as blue lines. The ribbons and amino acid side chains are colored in yellow, blue, and gray for the 49-kDa (NDUFS2), PSST (NDUFS7), and ND4 subunits, respectively.

However, Z-FPM only formed hydrophobic interactions (Figure 3B), lacking additional stabilization via hydrogen bonding. This could be one of the reasons for the worsened scores that were predicted. Interestingly, the proposed binding mode of Z-FPM is inverted with respect to the ester and pyrazole groups.

Taken together, these docking results suggest that there might be an isomerism-dependent activity cliff at the NADH:ubiquinone oxidoreductase of E-/Z-FPM. Thus, from docking-based analysis, probably only E-FPM is assumed to have specific inhibitory effects at CI, whereas its Z-isomer would be less active or inactive.

To confirm this hypothesis in a toxicologically relevant test system, the neural crest cell migration assay (cMINC) was used.^70^ The assay allows direct readouts on viability and cell function (migration). Moreover, oxygen consumption of neural crest cells can be measured as a specific end point for mitochondrial electron transport chain inhibition. Although the oxygen consumption rate assay represents a functional test, a sequential injection of specific substrates and inhibitors (see Section 2.7) allows to derive a specific pattern for CI-inhibition as it has been shown previously.^29,75,76^

In a previous study, we had observed that the racemic mixture, E-/Z-FPM, specifically reduced NCC function with a BMC_10_ (V) of 6.6 μM and a BMC_25_ (M) of 3.4 μM (ratio 1.9).^29^ Our new data show that E-FPM had a more potent effect (nM-range) than the racemic mixture. Conversely, Z-FPM only showed an effect in the higher μM-range (Figure 4A and B), and this effect was based on unspecific cytotoxicity (ratio <1.3). NCC migration was not specifically affected.

**Figure 4:**
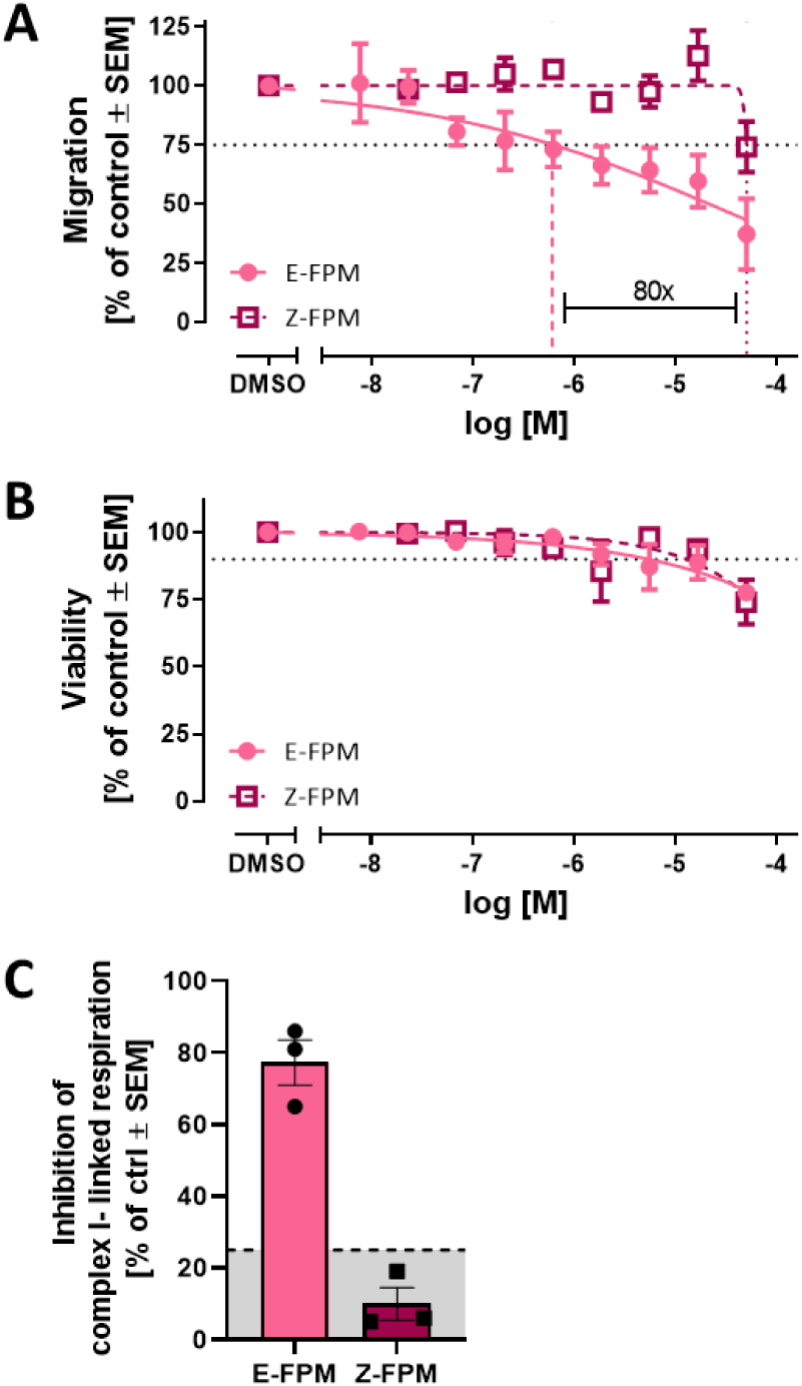
Effect of Fenpyroximate isomers on neural crest cells (NCC) (A) migration and (B) viability. Data are expressed as means ± SEM from at least three independent experiments. They are shown relative to the solvent control (0.1% DMSO). Horizontal dotted lines at (A) 75% and (B) 90% indicate relevant threshold concentrations. Vertical lines indicate their respective benchmark concentrations (BMC_25_ of migration and BMC_10_ of viability). (C) Inhibition of complex I-linked respiration (as % of control) was assessed in permeabilized cells at 10 min after treatment with the Fenpyroximate isomers. Specific inhibition of mitochondrial complex I has been proven by the assay validation.^29,75^ The exposure concentration was based on the BMC_10_ (V) of E-FPM (5 μM; see B). The gray area indicates the nonsignificant range of the assay (≤25% change). Data are expressed as means ± SEM from three independent experiments. E-FPM: E-Fenpyroximate. Z-FPM: Z-Fenpyroximate.

Thus, the BMC_25_ NCC migration assay data confirmed the presence of an isomerism-dependent activity cliff, showing an 80-fold difference of E- to Z-FPM (Table 2).

**Table 2:** Benchmark Concentrations 10 of Viability (BMC_10_ (V), i.e., Highest Noncytotoxic Concentration) and 25 of Migration (BMC_25_ (M), i.e., Threshold for Migration Impairment) of E-/Z-Fenpyroximate

	BMC_10_ (V)	BMC_25_ (M)	Ratio
**E-Fenpyroximate**	5 μM	0.6 μM	7.5
**Z-Fenpyroximate**	15 μM	>50 μM	<1

The cMINC assay represents a test method linked to key neurodevelopmental processes.^29,83,84^ It has a broad phenotypic readout, but does not inform on mechanisms. Therefore, we additionally used a test method that directly assesses CI function. The assay was performed in NCCs and used mitochondrial complex-specific substrates and inhibitors of CI as previously described.^29,75^ At the tested concentration (5 μM, based on BMC_10_ (V) of E-FPM), E-Fenpyroximate inhibited mitochondrial complex I, whereas Z-Fenpyroximate did not show specific inhibitory effects (Figure 4C). While these results indicate a reduction in mitochondrial respiration consistent with complex I inhibition, contributions from other mitochondrial or ETC components cannot be fully excluded.

These clear differences in two in vitro assays (cMINC, CI inhibition) corroborated the docking-based prediction, indicating higher toxicity of the E-isomer compared to the Z-form of Fenpyroximate. Moreover, this case study clearly highlights the importance of consideration of stereochemistry and isomerism in toxicological modeling, which supports integration of computational tools like molecular docking to capture such fine-grained structure–activity relationships.

#### 3.2.2 Combination of Consensus Scoring and In Vitro Assay Results at CIII

In general, classical empirical scoring functions are developed to estimate experimentally derived binding affinities.^32,85,86^ Thus, we assessed the extent to which consensus scoring and individual scoring functions aligned with experimental results, coming from an in vitro assay that measured mitochondrial complex II and III activity simultaneously (succinate cytochrome c (SCR) assay).^25,87^

Therefore, we evaluated which scoring function or consensus approach would have the best agreement with experimentally derived activities for a subset of 7 compounds (famoxadone, pyraclostrobin, trifloxystrobin, fluoxastrobin, azoxystrobin, kresoxim-methyl, and fenamidone) that specifically inhibit CIII (Table 3). Notably, one of the default scoring functions of GOLD, the ChemPLP Fitness function, showed the highest rank correlation with SCR assay activities (Spearman *r*: 0.89), indicating the best performance for this specific outcome. However, while the ChemPLP Fitness score and the ECR score showed essentially the same linear correlation to experimental outcomes (Pearson *r*: 0.82), their rank-based agreement diverged slightly, suggesting subtle differences in relative prioritization of ligands. The differences in ordering concerned mainly the compounds having low to medium activity, but they agreed on the most potent compounds, pyraclostrobin and famoxadone (Figure 5).

**Table 3:** Spearman Rank Correlations of Scoring Functions, Binding Energy Calculations, and Consensus Approaches to In Vitro Assay Results

Scoring Function	Spearman Correlation to SCR pIC50	Pearson Correlation to SCR pIC50
Glide IFDScore	0.21	–0.20
MMGBSA dG Bind (GOLD)	0.21	–0.32
Glide docking score	0.29	–0.42
MBAE Del Total Energy-S-OPLS (GOLD)	0.32	–0.33
MBAE ASET Total Energy-S-OPLS (GOLD)	0.39	–0.24
GOLD ChemScore	0.39	0.45
MBAE Del Total Energy-S-OPLS (GOLD)	0.43	–0.45
MBAE ASET Total Energy-S-OPLS (Glide)	0.54	–0.33
MMGBSA dG Bind (Glide)	0.57	–0.64
GOLD GoldScore	0.64	0.76
aggregated_rank	0.86	n.d.
ECR	0.86	0.82
GOLD ChemPLP Score	0.89	0.82

**Figure 5:**
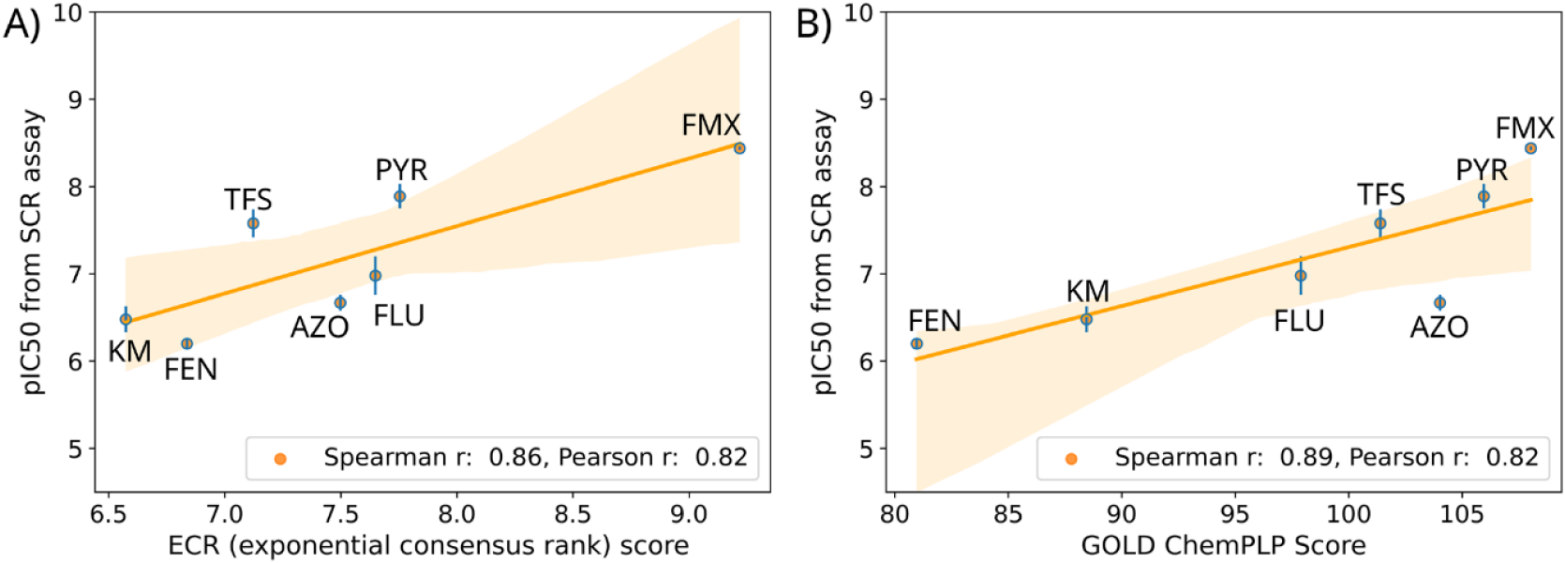
Regression plots of 7 CIII-inhibitors displaying the linear correlation of (A) the consensus ECR scores and (B) the ChemPLP Fitness scoring functions from GOLD to pIC50 values from an SCR assay, that was reported previously.^25,87^ The regression lines are shown in orange and the according 95%-confidence interval is displayed as light-orange band. Standard deviations from the pIC50 values are shown as blue vertical lines. ECR: Exponential consensus rank. KM: Kresoxim-methyl. FEN: Fenamidone. TFS: Trifloxystrobin. AZO: Azoxystrobin. FLU: Fluoxastrobin. PYR: Pyraclostrobin. FMX: Famoxadone.

These observations are also reflected in regression plots where the linear predictivity of ECR and ChemPLP Fitness score to experimentally derived pIC50 results is displayed (Figure 5). Here, the ECR approach assigns a significantly higher score to Famoxadone (pIC50 of 8.5), since it was ranked consistently high across the individual scoring functions (Figure 5A). On the other hand, the ChemPLP Fitness scores of all compounds are in a narrower range (Figure 5B). Therefore, differences between relative activities in compounds are less pronounced using solely one score here. In this case, both consensus approaches performed equally well for this rank-based prioritization task according to the Spearman *r* (both Spearman *r*: 0.86). Both strategies aligned on the highest and lowest ranked compounds, but they diverged in rankings of compounds having medium activity.

In the context of toxicology, consensus scoring-derived compound prioritization has the potential to select compounds that would need follow-up testing in a tiered approach, as we previously exemplified for E- and Z-Fenpyroximate.

### 3.3 Protein–Ligand Interaction Analysis

In addition to relative rankings and consensus scoring approaches, docking results are often utilized to study the underlying molecular interactions causing potential differences in target protein binding. Herein, representative docking poses from both Glide and GOLD were minimized using the MM-GBSA Prime tool.^52^ Subsequently, the minimized protein–ligand complexes were analyzed using PLIP.^60^ Merging the interactions coming from both docking outputs per ligand results in a consensus interaction profile of molecular interactions. This consensus interaction fingerprint aims to increase confidence in the predicted interaction profiles by combining the results from different docking placement algorithms.

#### 3.3.1 Consensus Interactions at Mitochondrial Complex I

The Qd (deep quinone) binding site of CI, represents the major inhibitor and quinone binding site and connects the mitochondrial membrane to the catalytic core of the complex.^39^ The overall interaction profile of the CI-inhibitors, that were docked herein, predominantly reports hydrophobic contacts (Figure S5A), for example, to the side chain of Phe110 in the PSST subunit. Within the coupling mechanism of mammalian CI, binding of ligands can affect the conformation of the 49-kDa loop (chain Q in PDB-ID 5xtd),^26^ where most of the key interaction residues are located. Among the amino acids that were previously reported to be of importance for binding the headgroup of quinone are His92 and Tyr141 (His59 and Tyr108 in ovine complex PDB-ID 6zkc).^39^ Those two residues are also known to be the key amino acids that coordinate the inhibitors rotenone and piericidin A.^39,41,88^ In our study, Tyr141 represented a minor role in ligand binding, probably because the side chain conformations were not sufficiently sampled, although an induced fit protocol was applied (Figure S7). Nevertheless, His92 contributed to hydrophobic interactions and hydrogen bonds, which are also reflected in the consensus interaction fingerprint (Figures S5A, S6A).

The consensus interaction fingerprint was generated by combining the predicted, unique interactions of the ligands that were docked using two different docking algorithms (Figure S6). This results in an interaction fingerprint of 21-bit length. Those fingerprints can be utilized to compare the similarity of the interaction profiles, for example, in terms of pairwise Tanimoto similarities (Figure S5B).

Regarding the consensus interaction fingerprints of the previously discussed case study compounds, E- and Z-Fenpyroximate, they do not overlap at all since their proposed binding modes were very different. Common chemical fingerprints cannot sufficiently capture the difference between those geometric isomers, whereas interaction fingerprints from docking and different scoring behavior captured differences herein (Figure S5B and C).

It is worth highlighting that CI-inhibitors having a biguanide substructure (metformin, phenformin, lixumistat) might target a different, lower affinity binding site of CI within the membrane domain of the ND4 subunit.^39^ However, this binding site was not accessible in the structure that was used for the underlying study, as it presents a different conformational state (i.e., deactive conformation according to Kampjut and Sazanov 2020,^39^ open state in ovine PDB-ID 6zkm,^39^ native conformation, closed state in human PDB-ID 5xtd^26^). This hypothesis was strengthened by the observation that metformin poses did not generate a consensus fingerprint. Also, the fingerprints for phenformin and lixumistat only consist of unspecific hydrophobic contacts to His92 and Ala90 (phenformin) (Figure S6A).

#### 3.3.2 Consensus Interactions at Mitochondrial Complex III

PLIP analysis revealed that the following residues contributed the most to the binding of CIII-inhibitors at the human Qo (quinol oxidation) binding site (5xte chain V): Phe274, Phe128, Ile146, Pro270, Glu271, and Tyr145 (Figure 6A). Overall, the majority of interactions are hydrophobic, which aligns with the fact that the Qo binding site is known to be lipophilic to accommodate also endogenous lipophilic quinone substrates.^44^

**Figure 6:**
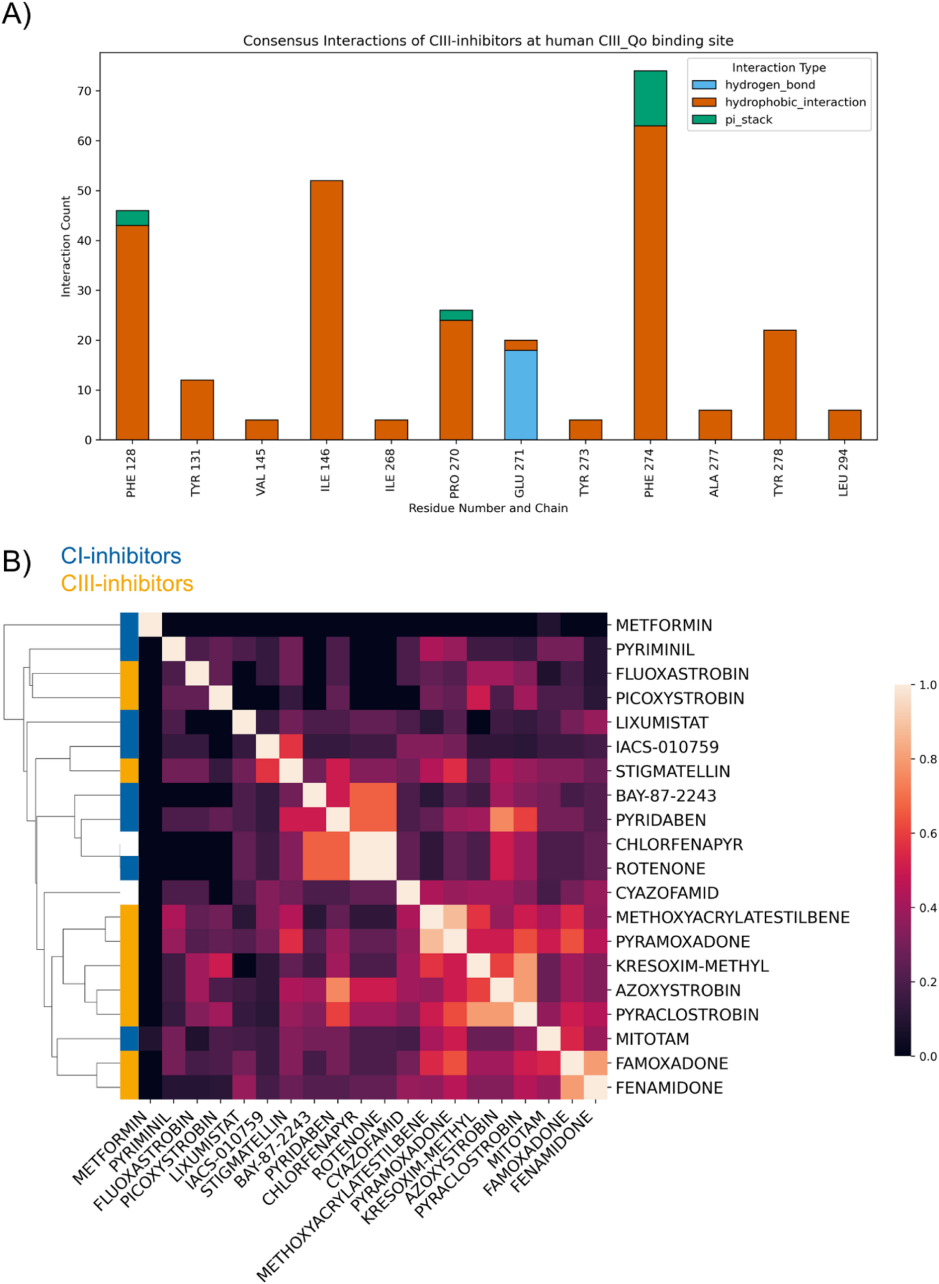
Consensus interaction profiles at the CIII-Qo binding site. (A) Stacked bar plot of interactions formed by CIII-inhibitors from docking into CIII. Hydrophobic contacts are colored in orange, hydrogen bonds in blue, pi-stacking in green. (B) Hierarchically clustered heatmap of the consensus interaction fingerprint generated from consensus docking and PLIP.^60^ Color intensity within the heatmap corresponds to Tanimoto Similarity of the interaction fingerprints, with lighter shades indicating higher similarity. The cluster bar on the left reflects the hierarchical organization of compounds by Tanimoto distance of consensus interactions, where uncouplers, CI- and CIII-inhibitors are labeled in white, blue, and yellow, respectively.

Phe274 additionally contributes to pi-stacking interactions, which have been reported in literature previously.^89^ Moreover, molecular interactions with Glu271 and Pro270 have also been identified as important.^25^ Especially hydrogen-bonding to the backbone of Glu271 is the common interaction for strobilurin fungicides and their toxophoric methoxy-acrylate-substructure (Figures S1A and S6B). However, also imidazolidine-like Qo-inhibitors (famoxadone, fenamidone, pyramoxadone) formed contacts with Glu271 (Figure S1B). Qo-/Qp-site inhibitors are also classified based on the conformational effect they induce in CIII. P_f_-type inhibitors like stigmatellin and famoxadone lead to immobilization or fixation of the Rieske iron–sulfur protein (ISP)-extrinsic domain, whereas P_m_-type inhibitors, such as azoxystrobin, rather induce the mobility of the ISP.^79^

It has been postulated that interactions of Qo-inhibitors with Tyr278, which is part of the ISP docking crater, might induce ISP mobility because the inhibitor’s interaction would hinder ISP binding.^90^ Most of the P_m_-type inhibitors (pyraclostrobin, picoxystrobin, kresoxim-methyl, methoxy-acrylate-stilbene) reported this interaction from the consensus interaction fingerprints. However, we also found P_f_-type inhibitors like famoxadone to interact with Tyr278. Nevertheless, stigmatellin did not form contacts to this residue, hence supporting the hypothesis of P_f_-type inhibitors leading to immobilization. Interestingly, P_f_-type inhibitors stigmatellin, famoxadone and fenamidone showed overall a different interaction pattern compared to P_m_-type (Figure 6B) inhibitors like azoxystrobin. Nevertheless, according to our study, the main difference in the interaction pattern of the P_f_-type inhibitors Fenamidone and Famoxadone seemed to be additional hydrophobic interactions to Ile268 and Tyr273, respectively.

Notably, the structure used for the underlying study represents the c position of the Rieske domain, i.e., the loading state of cytochrome c,^26^ which is probably stabilized by P_m_-type inhibitors.^91^ Another study used the bovine complex with stigmatellin (PDB-ID: 1pp9),^25,92^ representing the proximal position of the ISP head domain. In that study, His161, that ligates the iron cluster, has been identified as an important interactor for Qo-inhibitors,^25^ but it is probably more relevant for P_f_-type inhibitors. Since the focus of this study lay more on P_m_-type inhibitors, the choice for the human structure 5xte as crystal structure is regarded as plausible.

## 4 Discussion

Ligand binding to the main inhibitor binding sites of human mitochondrial complex I and III (CI and CIII) represents relevant molecular initiating events potentially leading to adverse outcomes linked to neuronal impairment.^27,29^ Herein, we applied two classical state-of-the-art molecular docking algorithms with an induced fit protocol and performed rescoring using different scoring functions and a set of binding energy calculations for 28 mitochondrial toxicants known to bind to either CI or CIII, or to neither of them (mitochondrial uncoupling compounds). Our findings support the utility of using consensus docking and scoring in a tiered approach for toxicological hazard characterization. In this setting, conventional QSAR or machine learning models that predict e.g., end points like mitochondrial toxicity,^9–14^ could be used as initial screening method to flag potentially problematic compounds. Subsequently, flagged compounds are subjected to consensus docking protocols, which can then be used further for prioritizing compounds for follow-up testing in in vitro systems, based on predicted relative binding affinities at CI and CIII. In this context, docking scores not only served as predictive features but also enabled mechanistic interpretation through analysis of interaction patterns. The correlation and complementarity with in vitro outcomes highlight the potential of molecular docking in predictive toxicology workflows such as ASPA (https://www.risk-hunt3r.eu/aspa/).^93,94^

Conformational flexibility of the CI- and CIII-binding sites was accounted for by applying an induced fit docking protocol. Upon induced fit docking (IFD) in Schrödinger, more relaxed energy cutoffs (reduced van der Waals (vdW) radii and increased Coulomb-vdW cutoff thresholds) are applied, while in addition, amino acids with bulky side chains are temporarily mutated to alanine to reduce steric clashes upon ligand placement. The use of IFD also enabled successful docking of all the ligands that were assessed herein, while rigid docking protocols skipped several ligands, e.g., due to energy filters within the internal Glide pose filtering process. Subsequently, after ligand placement, the lowest energy conformation of the protein is predicted for each generated binding pose, which is further scored by the IFDScore, which combines GlideScore and Prime Energy.^54^ However, while amino acid side chain rotamers are considered, movements of the protein backbone are rather neglected. Also, the docking protocol within GOLD only considers rotations of amino acid side chains. Unfortunately, we observed that the sampling of side chains was still not sufficiently modeled in all cases. For example, in CI Tyr141 was only rarely contacted, probably due to limited torsional sampling. Furthermore, in CIII, we had to modify the conformation of Glu271 before starting the IFD protocols (Figure S1) in a way that appropriate binding modes could be generated, as respective steric clashes were not resolved within IFD. Another strategy to account for protein flexibility would be ensemble docking, using different conformations that were experimentally resolved in the form of crystal structures, or from intermediate states of MD simulation trajectories, which was out of the scope for this study.

A docking score-based classification threshold using the intersection point between the distributions of binders and nonbinders can be applied for virtual screening purposes. Nevertheless, this approach works best when several thousand ligands with specific activity labels are available,^21,77^ which was not applicable for this study. The majority of activity labels we found upon literature search were on the level of mitochondrial toxicity, but not on the specific mode of action within the complexes of the electron transport chain. However, a preliminary IFDScore-based classification threshold for CI, that was applied for distinguishing CI- and non-CI-inhibitors, could be derived. Given the limited number of compounds (28 cross-docked ligands: 25 CI- and CIII-inhibitors and 3 uncouplers) that were evaluated herein, further validation of more ligands would be needed to generalize this threshold for use in virtual screening challenges. The docking protocol presented herein is computationally quite expensive and thus probably not suitable for fast screening of large compound libraries. Hence, the current study rather represents a refinement step for compound prioritization for follow-up in vitro testing, whereby the thresholds from individual scoring functions can serve as guidance points rather than absolute decision thresholds.

It should be emphasized that the performance of docking scoring functions is often target dependent, hence, validation upon development is essential. Within this study, we have focused on classical state-of-the-art scoring functions since they have been broadly validated for virtual screening purposes.^32,85^ (Semi)­empirical docking scores, as applied herein, are calculated by adding energy contributions coming from inter- and intramolecular contacts and geometric influence. They were trained to estimate experimental binding affinities by optimizing the weights of contribution of the individual energy terms.^86^ Hence, molecules with many functional groups that could contribute to favorable interactions represent a ligand-based bias,^95^ while increased van der Waals contact areas from large binding sites might contribute to a protein-based scoring bias.^95,96^ In particular, MitoTam, a mitochondria-targeting tamoxifen analogue tagged with a triphenylphosphonium cation, which is anticipated to target CI,^97^ has many functional groups that can increase the interaction count and, thus, bias the scoring from the ligand to a certain extent. This was also reflected by the fact that, from consensus scoring at CIII, MitoTam was ranked highly too. In general, cationic groups can lead to increased mitochondrial targeting since the proton gradient across the inner mitochondrial membrane facilitates drug delivery to the ETC targets.^97^

In addition to ligand-based bias in docking scoring functions, protein-based bias is also of relevance. For example, the IFDScore^54^ is a combination of Prime Energy^52,55^ and Glide Score,^32^ hence, the size of the protein drastically influences the scoring. Since for CI, more protein chains were retained for the docking study, as for CIII, IFDScores of CI are more negative simply because the size of the protein influences this score (Prime Energy). In this study, we observed that the IFDScore was able to differentiate between CI-inhibitors and non-CI-inhibitors from docking into CI (Figure 1), which was, however, not the case from docking into CIII (Figure S3). The hypothesis that scoring functions are dependent on the protein structures has been described previously.^96,98,99^ One method to compensate for target dependence of scoring functions, especially in reverse docking studies, would be docking score normalization.^96^ In this study, we chose rank-based consensus scoring approaches to overcome specific protein-based offsets and to be able to compare the relative rankings of multiple scoring functions between proteins. By deriving relative ranks (aggregated rank), and assuming an exponential distribution (ECR)^59^ over the rankings of each scoring function, we aimed to produce more robust docking results by combining multiple docking programs, while overcoming the issue of different units, scales and ranking directions (i.e., more negative values for Glide indicate better scores, whereas for GOLD Fitness function, it is inverted (the higher the better)). We found that both consensus ranking techniques were highly correlated with each other in both complexes (Figure 2), and they had the same performance regarding the rank-based prioritization at CIII, hence aligning with the ranking derived from the SCR assay (Spearman *r*: 0.86; see Figure 5A, Table 3). Additionally, the consensus strategies were able to separate the density curves of CIII- and non-CIII-inhibitors (CI-inhibitors and uncouplers). This was also the case for two scoring functions from GOLD (ChemPLP Score and GoldScore) and the MMGBSA dG Bind energies from GOLD generated docking poses (MMGBSA dG Bind (GOLD)) at CIII (Figure S3). Contrarily, at CI, only the IFDScore reported the highest density of CI-inhibitors at low ranks, whereas the consensus scoring strategies reported overlapping density curves (Figure S2). Despite that, the consensus scoring approaches reported differences between E-/Z-Fenpyroximate by capturing that the E-isomer was rewarded over the Z-form by multiple scoring functions. Thus, the problem of target-dependent scoring functions was not fully solved by consensus scoring strategies for differentiating binders and nonbinders. For this task, according to our results assessment of individual scoring functions worked best (Table S2).

As outlined in the introduction, machine learning-based models rarely capture stereoisomerism. The scoring results obtained for E-/Z-Fenpyroximate showcase the importance of considering isomerism in a toxicological context. Here, we compared each scoring function in terms of relative preferential binding affinity to human CI of the two isomers. This represents a majority vote approach, where E-Fenpyroximate was predicted to be more active than its Z-form (Table 3). In concordance with the docking-based predictions, we presented data from an oxygen consumption test for validation of the specific inhibition at CI by E-Fenpyroximate (Figure 4C). Moreover, measurable effects upon migration and cytotoxicity (Figure 4A and B, cMINC assay^70^) indicate potential impairments of key neurodevelopmental processes. The docking protocol simulates the binding of E- and Z-Fenpyroximate at the target site of CI and we assumed that the binding affinity is correlated to the inhibitory effects that are measured by the functional assays (Figure 4). However, the binding affinity estimates and scoring functions were validated against a functional readout rather than a thermodynamic binding assay, and therefore may not fully capture the true binding energetics. In this context, other effects beyond binding energetics, including assay-dependent effects, or binding to other competing (mitochondrial) targets could also influence the observed functional effects and the interpretation of the calculations. Nevertheless, this example showcases the complementarity of in silico and in vitro test systems in next-generation toxicological risk assessment.

In case of compound prioritization, correct rankings are of higher importance and usually perform better than a linear correlation between docking scores and biological assay-derived pIC50 values. Accordingly, evaluation of Spearman coefficients instead of Pearson correlations represents a more suitable metric. Among the scoring functions tested, the scoring function of GOLD, ChemPLP Fitness function reported the most consistent ranking concordance with in vitro data (Spearman *r*: 0.89), followed by the two consensus scoring strategies, aggregated ranking and ECR (both Spearman *r*: 0.86). It might well be that the ChemPLP Score works particularly well for this set of 7 CIII-inhibitors, while the consensus scores rather reflect the average performance over all scoring functions, including their potential intrinsic bias. As with every consensus-based strategy, the predictive confidence is increased when multiple methods converge on the same outcome.

This study builds on prior efforts by incorporating consensus docking and scoring strategies,^59,100^ while applying induced fit protocols, which have not been extensively evaluated in previous computational toxicology pipelines. By using two different docking algorithms that apply distinct ligand placement methods—exhaustive search for Glide and a genetic algorithm in GOLD,^32,33^ we aimed to strengthen the reliability of the respective interaction fingerprint that is derived. Only interactions that were present in both binding poses were used for the consensus protein–ligand interaction fingerprint. Nevertheless, this merging step also reduces the number of unique combinations of interaction type and protein residues, consequently leading to a shorter fingerprint. Therefore, a direct comparison of the 21-bit protein–ligand interaction fingerprint with 166-bit MACCS keys is not possible, which led us to compare the Tanimoto similarity matrices instead (Figure S5B and C). Given that the consensus interaction fingerprints from E- and Z-Fenpyroximate do not overlap (Figure S6A), the isomeric pair was separated by this fingerprint in the similarity matrix (Figure S5B), unlike in the case of MACCS keys (or other chemical descriptors), where they would remain indistinguishable from each other (Figure S5C). MACCS keys and consensus interaction fingerprints could also be combined, so that both specific protein–ligand interaction profiles and description of chemical features are captured and can be used for further analysis. A similar combination has been successfully applied for G-protein-coupled receptors previously.^101^

However, the merging step in generating consensus interaction fingerprints coming from consensus docking introduces the risk of losing an important interaction when suboptimal poses are combined. In this context, we observed that for some compounds, no common interactions are left, thus generating no consensus interaction fingerprint for the target. This could also be interpreted in a way that those compounds are unlikely to bind at the specific target binding site and in future studies other binding sites could be explored, e.g., with blind docking approaches. This was suspected for example for metformin, which did not generate a consensus fingerprint from docking into the Qd site of CI and, moreover, exhibited worsened scores compared to other CI-inhibitors. This suggests that a combination of interactions and scoring functions is relevant for studies on molecular initiating events, as also observed in a nuclear receptor case study in the recently developed DockTox application.^23^

In general, Qo-inhibitors competitively block access of quinol to the Qo binding site (also known as Qp), hence inhibiting electron transport (ET).^102^ Moreover, there are subtypes of Qo-site inhibitors that are classified based on distinct induced structural changes of the cytochrome bc1 complex upon ligand binding: P_f_-type inhibitors, like stigmatellin and famoxadone lead to immobilization or fixation of the ISP-extrinsic domain, whereas P_m_-type inhibitors, such as azoxystrobin, rather induce the mobility of the ISP.^44,79^ From interaction fingerprint-based clustering at the CIII-Qo binding site, we also observed clustering of those inhibitor subtypes (Figure 6B).

An important limitation of docking is the assumption that the compounds are already present at the binding site, thus neglecting any prior processes such as exposure, absorption, distribution, and metabolism. Regarding metabolism, it would be possible to include metabolites in the docking study as well.^103–105^ In future studies, approaches combining both molecular docking-derived information and machine learning have the potential to leverage the maximum information for predictive toxicology applications.

## 5 Conclusions

Herein, we performed a consensus docking study while applying an induced fit docking protocol. Preliminary scoring-based classifications at human mitochondrial CI and CIII were introduced, that could, to a certain extent, distinguish between CI- and CIII-inhibitors. Furthermore, consensus scoring approaches that combine multiple scoring functions and binding energy calculations have been shown to have good ranking correlations to experimentally derived pIC50 values at CIII. Also, we have emphasized that consensus scoring strategies that are presented herein, have the potential to compensate for some target-dependent biases, aiming for more reliable docking-based predictions. Moreover, a relative prioritization of a geometric isomer of the CI-inhibitor Fenpyroximate was evaluated using multiple scoring functions and predicted binding modes. The difference of isomerism on the double bond probably would not have been found in classical machine learning methods. In a tiered approach, we followed up with two suitable in vitro assay systems that confirmed the isomerism-dependent activity cliff. Additionally, we demonstrated the concept of consensus interaction fingerprints, that aim to gain confidence in the predicted protein–ligand interaction profiles and can be used to capture subtle differences in subtypes of binders.

## Supplementary Material





## Notes

Table S1: chemical structures and SMILES including references for functional and binding assays (XLSX)
Table S2: performance metrics of scoring-based classification thresholds; Figure S1: binding modes and redocking results at CIII; Figure S2: KDE plots of rankings from docking into CI; Figure S3: KDE plots of rankings from docking into CIII; Figure S4: KDE plots and confusion matrices of intersection points related to Table S2; Figure S5: interaction profiles at CI; Figure S6: consensus interaction fingerprint profiles; Figure S7: redocking results at CI (PDF)

## Abbreviations

BAC: balanced accuracy
BMC_10_ (V): benchmark concentration 10, i.e., highest noncytotoxic concentration
BMC_25_ (M): benchmark concentration 25, i.e., threshold for migration impairment
CI: mitochondrial complex I (NADH:ubiquinone oxidoreductase)
CIII: mitochondrial complex III (cytochrome bc_1_ complex)
cMINC: neural crest cell migration assay
ECR: exponential consensus rank
E-/Z-FPM: E-/Z-Fenpyroximate
IFD: induced fit docking
KDE: kernel density estimate
MCC: Matthews Correlation Coefficient
NCC: neural crest cells
Qd: deep quinol/quinone binding site in NADH:ubiquinone oxidoreductase (ROT1)
Qo: quinol oxidation site in cytochrome bc_1_ complex
QSAR: Quantitative Structure–Activity Relationship

## References

[1] Krewski D., Acosta D., Andersen M., Anderson H., Bailar J. C., Boekelheide K., Brent R., Charnley G., Cheung V. G., Green S., Kelsey K. T. (2010). Staff of Committee on
Toxicity Testing and Assessment of Environmental Agents. Toxicity
Testing in the 21st Century: A Vision and a Strategy. J. Toxicol. Environ. Health, Part B.

[2] Krewski D., Andersen M. E., Tyshenko M. G., Krishnan K., Hartung T., Boekelheide K., Wambaugh J. F., Jones D., Whelan M., Thomas R., Yauk C., Barton-Maclaren T., Cote I. (2020). Toxicity Testing in
the 21st Century: Progress in the Past Decade
and Future Perspectives. Arch. Toxicol..

[3] EMA ICH M7(R2) Guideline on Assessment and Control of DNA Reactive (Mutagenic) Impurities in Pharmaceuticals to Limit Potential Carcinogenic Risk; European Medicines Agency, 2023.

[4] Dimitrov S.
D., Diderich R., Sobanski T., Pavlov T. S., Chankov G. V., Chapkanov A. S., Karakolev Y. H., Temelkov S. G., Vasilev R. A., Gerova K. D., Kuseva C. D., Todorova N. D., Mehmed A. M., Rasenberg M., Mekenyan O. G. (2016). QSAR Toolbox – Workflow and
Major Functionalities. SAR QSAR Environ. Res..

[5] Myatt G. J., Ahlberg E., Akahori Y., Allen D., Amberg A., Anger L. T., Aptula A., Auerbach S., Beilke L., Bellion P. (2018). In Silico Toxicology Protocols. Regul. Toxicol. Pharmacol.

[6] OECD OECD QSAR Toolbox V4. 5; OECD, 2021.

[7] Dykens J. A., Will Y. (2007). The Significance of Mitochondrial
Toxicity Testing in Drug Development. Drug Discovery
Today.

[8] Meyer J. N., Hartman J. H., Mello D. F. (2018). Mitochondrial Toxicity. Toxicol. Sci..

[9] Zhang H., Chen Q.-Y., Xiang M.-L., Ma C.-Y., Huang Q., Yang S.-Y. (2009). In silico prediction
of mitochondrial toxicity by using
GA-CG-SVM approach. Toxicol. In Vitro.

[10] Hemmerich J., Troger F., Füzi B., Ecker F. G. (2020). Using Machine Learning
Methods and Structural Alerts for Prediction of Mitochondrial Toxicity. Mol. Inf..

[11] Bringezu F., Carlos Gómez-Tamayo J., Pastor M. (2021). Ensemble Prediction
of Mitochondrial Toxicity Using Machine Learning Technology. Comput. Toxicol..

[12] Seal S., Carreras-Puigvert J., Trapotsi M.-A., Yang H., Spjuth O., Bender A. (2022). Integrating Cell Morphology with Gene Expression and
Chemical Structure to Aid Mitochondrial Toxicity Detection. Commun. Biol..

[13] Igarashi Y., Kojima R., Matsumoto S., Iwata H., Okuno Y., Yamada H. (2024). Developing a GNN-Based
AI Model to Predict Mitochondrial
Toxicity Using the Bagging Method. J. Toxicol
Sci..

[14] Garcia
de Lomana M., Marin Zapata P. A., Montanari F. (2023). Predicting
the Mitochondrial Toxicity of Small Molecules: Insights from Mechanistic
Assays and Cell Painting Data. Chem. Res. Toxicol..

[15] Tang W., Liu W., Wang Z., Hong H., Chen J. (2022). Machine Learning Models
on Chemical Inhibitors of Mitochondrial Electron Transport Chain. J. Hazard. Mater..

[16] Seal S., Mahale M., García-Ortegón M., Joshi C. K., Hosseini-Gerami L., Beatson A., Greenig M., Shekhar M., Patra A., Weis C., Mehrjou A., Badré A., Paisley B., Lowe R., Singh S., Shah F., Johannesson B., Williams D., Rouquie D., Clevert D.-A., Schwab P., Richmond N., Nicolaou C. A., Gonzalez R. J., Naven R., Schramm C., Vidler L. R., Mansouri K., Walters W. P., Wilk D. D., Spjuth O., Carpenter A. E., Bender A. (2025). Machine Learning for Toxicity Prediction
Using Chemical Structures: Pillars for Success in the Real World. Chem. Res. Toxicol..

[17] Das J., Rao C. V. L., Sastry T. V. R. S., Roshaiah M., Sankar P. G., Khadeer A., Kumar M. S., Mallik A., Selvakumar N., Iqbal J., Trehan S. (2005). Effects of
Positional and Geometrical
Isomerism on the Biological Activity of Some Novel Oxazolidinones. Bioorg. Med. Chem. Lett..

[18] Vargesson N. (2015). Thalidomide-Induced
Teratogenesis: History and Mechanisms. Birth
Defects Res. C Embryo Today.

[19] Kaneko H., Korenaga R., Nakamura R., Kawai S., Ando T., Shiroishi M. (2024). Binding Characteristics
of the Doxepin E/Z-Isomers
to the Histamine H1 Receptor Revealed by Receptor-Bound Ligand Analysis
and Molecular Dynamics Study. J. Mol. Recognit..

[20] Smith S. W. (2009). Chiral
Toxicology: It’s the Same Thing··· Only Different. Toxicol. Sci..

[21] Trisciuzzi D., Alberga D., Mansouri K., Judson R., Cellamare S., Catto M., Carotti A., Benfenati E., Novellino E., Mangiatordi G. F., Nicolotti O. (2015). Docking-Based
Classification Models for Exploratory Toxicology Studies on High-Quality
Estrogenic Experimental Data. Future Med. Chem..

[22] Kan H.-L., Tung C.-W., Chang S.-E., Lin Y.-C. (2022). In Silico Prediction
of Parkinsonian Motor Deficits-Related Neurotoxicants Based on the
Adverse Outcome Pathway Concept. Arch. Toxicol..

[23] Ortega-Vallbona R., Talavera-Cortés D., Carpio L. E., Coto
Palacio J., Roncaglioni A., Garcia De Lomana M., Gadaleta D., Benfenati E., Gozalbes R., Serrano-Candelas E. (2025). DockTox: Targeting
Molecular Initiating Events in Organ Toxicity through Molecular Docking. Toxicology.

[24] Troger F., Delp J., Funke M., van der Stel W., Colas C., Leist M., van de Water B., Ecker G. F. (2020). Identification of Mitochondrial Toxicants by Combined
in Silico and in Vitro Studies – A Structure-Based View on
the Adverse Outcome Pathway. Comput. Toxicol..

[25] Rosell-Hidalgo A., Moore A. L., Ghafourian T. (2023). Prediction
of Drug-Induced Mitochondrial
Dysfunction Using Succinate-Cytochrome c Reductase Activity, QSAR
and Molecular Docking. Toxicology.

[26] Guo R., Zong S., Wu M., Gu J., Yang M. (2017). Architecture
of Human Mitochondrial Respiratory Megacomplex I_2_III_2_IV_2_. Cell.

[27] AOP-Wiki AOP 3. Mitochondrial dysfunction and Neurotoxicity, AOP-Wiki release 2.7. https://aopwiki.org/aops/3. (Accessed 20–06–2025).

[28] Terron A., Bal-Price A., Paini A., Monnet-Tschudi F., Bennekou S. H., Leist M., Schildknecht S. (2018). EFSA WG EPI1Members.
An Adverse Outcome Pathway for Parkinsonian Motor Deficits Associated
with Mitochondrial Complex I Inhibition. Arch.
Toxicol..

[29] Magel V., Blum J., Dolde X., Leisner H., Grillberger K., Khalidi H., Gardner I., Ecker G. F., Pallocca G., Dreser N., Leist M. (2024). Inhibition
of Neural Crest Cell Migration
by Strobilurin Fungicides and Other Mitochondrial Toxicants. Cells.

[30] Urra F. A., Muñoz F., Lovy A., Cárdenas C. (2017). The Mitochondrial
Complex­(I)­Ty of Cancer. Front. Oncol..

[31] Vercellino I., Sazanov L. A. (2022). The Assembly, Regulation and Function of the Mitochondrial
Respiratory Chain. Nat. Rev. Mol. Cell Biol..

[32] Friesner R. A., Banks J. L., Murphy R. B., Halgren T. A., Klicic J. J., Mainz D. T., Repasky M. P., Knoll E. H., Shelley M., Perry J. K., Shaw D. E., Francis P., Shenkin P. S. (2004). Glide:
A New Approach for Rapid, Accurate Docking and Scoring. 1. Method
and Assessment of Docking Accuracy. J. Med.
Chem..

[33] Jones G., Willett P., Glen R. C., Leach A. R., Taylor R. (1997). Development
and Validation of a Genetic Algorithm for Flexible Docking. J. Mol. Biol..

[34] Mansouri K., Abdelaziz A., Rybacka A., Roncaglioni A., Tropsha A., Varnek A., Zakharov A., Worth A., Richard A. M., Grulke C. M., Trisciuzzi D., Fourches D., Horvath D., Benfenati E., Muratov E., Wedebye E. B., Grisoni F., Mangiatordi G. F., Incisivo G. M., Hong H., Ng H. W., Tetko I. V., Balabin I., Kancherla J., Shen J., Burton J., Nicklaus M., Cassotti M., Nikolov N. G., Nicolotti O., Andersson P. L., Zang Q., Politi R., Beger R. D., Todeschini R., Huang R., Farag S., Rosenberg S. A., Slavov S., Hu X., Judson R. S. (2016). CERAPP: Collaborative
Estrogen Receptor Activity Prediction Project. Environ. Health Perspect..

[35] Mansouri K., Kleinstreuer N., Abdelaziz A. M., Alberga D., Alves V. M., Andersson P. L., Andrade C. H., Bai F., Balabin I., Ballabio D., Benfenati E., Bhhatarai B., Boyer S., Chen J., Consonni V., Farag S., Fourches D., García-Sosa A.
T., Gramatica P., Grisoni F., Grulke C. M., Hong H., Horvath D., Hu X., Huang R., Jeliazkova N., Li J., Li X., Liu H., Manganelli S., Mangiatordi G. F., Maran U., Marcou G., Martin T., Muratov E., Nguyen D.-T., Nicolotti O., Nikolov N. G., Norinder U., Papa E., Petitjean M., Piir G., Pogodin P., Poroikov V., Qiao X., Richard A. M., Roncaglioni A., Ruiz P., Rupakheti C., Sakkiah S., Sangion A., Schramm K.-W., Selvaraj C., Shah I., Sild S., Sun L., Taboureau O., Tang Y., Tetko I. V., Todeschini R., Tong W., Trisciuzzi D., Tropsha A., Van Den
Driessche G., Varnek A., Wang Z., Wedebye E. B., Williams A. J., Xie H., Zakharov A. V., Zheng Z., Judson R. S. (2020). CoMPARA: Collaborative Modeling Project for Androgen
Receptor Activity. Environ. Health Perspect..

[36] Pradeep P., Povinelli R. J., White S., Merrill S. J. (2016). An Ensemble Model
of QSAR Tools for Regulatory Risk Assessment. J. Cheminf..

[37] Valsecchi C., Grisoni F., Consonni V., Ballabio D. (2020). Consensus versus Individual
QSARs in Classification: Comparison on a Large-Scale Case Study. J. Chem. Inf Model..

[38] Collins S. P., Mailloux B., Kulkarni S., Gagné M., Long A. S., Barton-Maclaren T. S. (2024). Development
and Application of Consensus
in Silico Models for Advancing High-Throughput Toxicological Predictions. Front. Pharmacol..

[39] Kampjut D., Sazanov L. A. (2020). The Coupling Mechanism
of Mammalian Respiratory Complex
I. Science.

[40] Gu J., Liu T., Guo R., Zhang L., Yang M. (2022). The Coupling Mechanism
of Mammalian Mitochondrial Complex I. Nat. Struct
Mol. Biol..

[41] Grba D. N., Chung I., Bridges H. R., Agip A.-N. A., Hirst J. (2023). Investigation
of Hydrated Channels and Proton Pathways in a High-Resolution Cryo-EM
Structure of Mammalian Complex I. Sci. Adv..

[42] Gutiérrez-Fernández J., Kaszuba K., Minhas G. S., Baradaran R., Tambalo M., Gallagher D. T., Sazanov L. A. (2020). Key Role of Quinone
in the Mechanism of Respiratory Complex I. Nat.
Commun..

[43] Bartlett D. W., Clough J. M., Godwin J. R., Hall A. A., Hamer M., Parr-Dobrzanski B. (2002). The Strobilurin Fungicides. Pest
Manage. Sci..

[44] Esser L., Quinn B., Li Y.-F., Zhang M., Elberry M., Yu L., Yu C.-A., Xia D. (2004). Crystallographic Studies of Quinol
Oxidation Site Inhibitors: A Modified Classification of Inhibitors
for the Cytochrome Bc1 Complex. J. Mol. Biol..

[45] Gao X., Wen X., Yu C., Esser L., Tsao S., Quinn B., Zhang L., Yu L., Xia D. (2002). The Crystal Structure
of Mitochondrial Cytochrome Bc1 in Complex with Famoxadone: The Role
of Aromatic–Aromatic Interaction in Inhibition. Biochemistry.

[46] Berman H. M., Westbrook J., Feng Z., Gilliland G., Bhat T. N., Weissig H., Shindyalov I. N., Bourne P. E. (2000). The Protein Data Bank. Nucleic
Acids Res..

[47] Schrödinger Release 2022–4: Protein Preparation Workflow; Epik, Prime, Schrödinger, LLC: New York, NY, 2022.

[48] Schrödinger Release 2022–4: LigPrep, Schrödinger, LLC, New York, NY, 2022.

[49] Madhavi
Sastry G., Adzhigirey M., Day T., Annabhimoju R., Sherman W. (2013). Protein and Ligand Preparation: Parameters, Protocols,
and Influence on Virtual Screening Enrichments. J. Comput. Aided Mol. Des..

[50] Lu C., Wu C., Ghoreishi D., Chen W., Wang L., Damm W., Ross G. A., Dahlgren M. K., Russell E., Bargen C. D. V. (2021). OPLS4: Improving Force Field Accuracy on Challenging
Regimes of Chemical Space. J. Chem. Theory Comput..

[51] Wohlwend D., Mérono L., Bucka S., Ritter K., Jessen H. J., Friedrich T. (2024). Structures of 3-Acetylpyridine Adenine
Dinucleotide
and ADP-Ribose Bound to the Electron Input Module of Respiratory Complex
I. Structure.

[52] Schrödinger Release 2022-4: Small Molecule Drug Discovery Prime; Schrödinger, LLC: New York, NY, 2022.

[53] Schrödinger Release 2022–4: Glide; Schrödinger, LLC: New York, NY, 2022.

[54] Sherman W., Day T., Jacobson M. P., Friesner R. A., Farid R. (2006). Novel Procedure for
Modeling Ligand/Receptor Induced Fit Effects. J. Med. Chem..

[55] Jacobson M. P., Friesner R. A., Xiang Z., Honig B. (2002). On the Role of the
Crystal Environment in Determining Protein Side-Chain Conformations. J. Mol. Biol..

[56] Li J., Abel R., Zhu K., Cao Y., Zhao S., Friesner R. A. (2011). The VSGB 2.0 Model: A next Generation
Energy Model
for High Resolution Protein Structure Modeling. Proteins: Struct., Funct., Bioinf..

[57] Mohamadi F., Richards N. G. J., Guida W. C., Liskamp R., Lipton M., Caufield C., Chang G., Hendrickson T., Still W. C. (1990). Macromodel—an Integrated Software
System for
Modeling Organic and Bioorganic Molecules Using Molecular Mechanics. J. Comput. Chem..

[58] Schrödinger Release 2022–4: MacroModel; Schrödinger, LLC: New York, NY, 2022.

[59] Palacio-Rodríguez K., Lans I., Cavasotto C. N., Cossio P. (2019). Exponential Consensus
Ranking Improves the Outcome in Docking and Receptor Ensemble Docking. Sci. Rep..

[60] Adasme M. F., Bolz S. N., Al-Fatlawi A., Schroeder M. (2022). Decomposing
Compounds Enables Reconstruction of Interaction Fingerprints for Structure-Based
Drug Screening. J. Cheminf..

[61] Hunter J. D. (2007). Matplotlib:
A 2D Graphics Environment. Comput. Sci. Eng..

[62] Waskom M. S. (2021). Statistical
Data Visualization. Joss.

[63] The Pandas Development Team. Pandas-Dev/Pandas; Pandas, 2025.

[64] Harris C. R., Millman K. J., van der Walt S. J., Gommers R., Virtanen P., Cournapeau D., Wieser E., Taylor J., Berg S., Smith N. J., Kern R., Picus M., Hoyer S., van Kerkwijk M. H., Brett M., Haldane A., Del Río J. F., Wiebe M., Peterson P., Gérard-Marchant P., Sheppard K., Reddy T., Weckesser W., Abbasi H., Gohlke C., Oliphant T. E. (2020). Array Programming
with NumPy. Nature.

[65] Virtanen P., Gommers R., Oliphant T. E., Haberland M., Reddy T., Cournapeau D., Burovski E., Peterson P., Weckesser W., Bright J. (2020). SciPy 1.0: Fundamental
Algorithms for Scientific Computing in Python. Nat. Methods.

[66] Pedregosa F., Varoquaux G., Gramfort A., Michel V., Thirion B., Grisel O., Blondel M., Prettenhofer P., Weiss R., Dubourg V. (2011). Scikit-Learn: Machine
Learning in Python. J. Mach. Learn. Res..

[67] Durant J.
L., Leland B. A., Henry D. R., Nourse J. G. (2002). Reoptimization of
MDL Keys for Use in Drug Discovery. J. Chem.
Inf. Comput. Sci..

[68] Schrödinger, LLC The PyMOL Molecular Graphics System, Version 2.5, Schrödinger, LLC, 2021.

[69] Mica Y., Lee G., Chambers S. M., Tomishima M. J., Studer L. (2013). Modeling Neural Crest
Induction, Melanocyte Specification, and Disease-Related Pigmentation
Defects in hESCs and Patient-Specific iPSCs. Cell Rep..

[70] Nyffeler J., Karreman C., Leisner H., Kim Y. J., Lee G., Waldmann T., Leist M. (2017). Design of a High-Throughput Human
Neural Crest Cell Migration Assay to Indicate Potential Developmental
Toxicants. ALTEX.

[71] Dolde X., Karreman C., Wiechers M., Schildknecht S., Leist M. (2021). Profiling of Human Neural Crest Chemoattractant
Activity as a Replacement
of Fetal Bovine Serum for In Vitro Chemotaxis Assays. Int. J. Mol. Sci..

[72] Stiegler N. V., Krug A. K., Matt F., Leist M. (2011). Assessment of Chemical-Induced
Impairment of Human Neurite Outgrowth by Multiparametric Live Cell
Imaging in High-Density Cultures. Toxicol. Sci..

[73] Krebs A., Nyffeler J., Rahnenführer J., Leist M. (2018). Normalization of Data
for Viability and Relative Cell Function Curves. ALTEX.

[74] Krebs A., Nyffeler J., Karreman C., Schmidt B. Z., Kappenberg F., Mellert J., Pallocca G., Pastor M., Rahnenführer J., Leist M. (2020). Determination of Benchmark Concentrations and Their Statistical Uncertainty
for Cytotoxicity Test Data and Functional in Vitro Assays. ALTEX.

[75] Delp J., Funke M., Rudolf F., Cediel A., Bennekou S. H., van der Stel W., Carta G., Jennings P., Toma C., Gardner I., van de Water B., Forsby A., Leist M. (2019). Development
of a Neurotoxicity Assay That Is Tuned to Detect Mitochondrial Toxicants. Arch. Toxicol..

[76] van
der Stel W., Carta G., Eakins J., Darici S., Delp J., Forsby A., Bennekou S. H., Gardner I., Leist M., Danen E. H. J., Walker P., van de Water B., Jennings P. (2020). Multiparametric Assessment of Mitochondrial Respiratory
Inhibition in HepG2 and RPTEC/TERT1 Cells Using a Panel of Mitochondrial
Targeting Agrochemicals. Arch. Toxicol..

[77] Jain S., Grandits M., Richter L., Ecker G. F. (2017). Structure Based
Classification for Bile Salt Export Pump (BSEP) Inhibitors Using Comparative
Structural Modeling of Human BSEP. J. Comput.
Aided Mol. Des..

[78] Esposti M. D., Ghelli A., Crimi M., Estornell E., Fato R., Lenaz G. (1993). Complex I and Complex III of Mitochondria
Have Common Inhibitors Acting as Ubiquinone Antagonists. Biochem. Biophys. Res. Commun..

[79] Esser L., Xia D. (2024). Mitochondrial Cytochrome
Bc1 Complex as Validated Drug Target: A
Structural Perspective. Trop. Med. Infect. Dis..

[80] Ghahremanpour M. M., Tirado-Rives J., Deshmukh M., Ippolito J. A., Zhang C.-H., Cabeza
de Vaca I., Liosi M.-E., Anderson K. S., Jorgensen W. L. (2020). Identification
of 14 Known Drugs as Inhibitors of the Main Protease of SARS-CoV-2. ACS Med. Chem. Lett..

[81] F. A. O. /. W. H. O. Pesticide Residues in Food 2017. In FAO Plant Production and Protection Paper. F. A. O. and W. H. O.: Rome, Italy, 2017; pp. 104.

[82] Murai M. (2020). Exploring
the Binding Pocket of Quinone/Inhibitors in Mitochondrial Respiratory
Complex I by Chemical Biology Approaches. Biosci.,
Biotechnol., Biochem..

[83] Bal-Price A., Hogberg H. T., Crofton K. M., Daneshian M., FitzGerald R. E., Fritsche E., Heinonen T., Hougaard
Bennekou S., Klima S., Piersma A. H., Sachana M., Shafer T. J., Terron A., Monnet-Tschudi F., Viviani B., Waldmann T., Westerink R. H. S., Wilks M. F., Witters H., Zurich M.-G., Leist M. (2018). Recommendation
on Test Readiness Criteria for New Approach Methods in Toxicology:
Exemplified for Developmental Neurotoxicity. ALTEX.

[84] Blum J., Masjosthusmann S., Bartmann K., Bendt F., Dolde X., Dönmez A., Förster N., Holzer A.-K., Hübenthal U., Keßel H. E. (2022). Establishment of a Human Cell-Based in Vitro
Battery to Assess Developmental Neurotoxicity Hazard of Chemicals. Chemosphere.

[85] Liebeschuetz J. W., Cole J. C., Korb O. (2012). Pose Prediction
and Virtual Screening
Performance of GOLD Scoring Functions in a Standardized Test. J. Comput. Aided Mol. Des..

[86] Li J., Fu A., Zhang L. (2019). An Overview
of Scoring Functions Used for Protein–Ligand
Interactions in Molecular Docking. Interdiscip
Sci. Comput. Life Sci..

[87] Wang F., Li H., Wang L., Yang W.-C., Wu J.-W., Yang G.-F. (2011). Design,
Syntheses, and Kinetic Evaluation of 3-(Phenylamino)­Oxazolidine-2,4-Diones
as Potent Cytochrome *Bc*1 Complex Inhibitors. Bioorg. Med. Chem..

[88] Bridges H. R., Fedor J. G., Blaza J. N., Di Luca A., Jussupow A., Jarman O. D., Wright J. J., Agip A.-N. A., Gamiz-Hernandez A. P., Roessler M. M., Kaila V. R. I., Hirst J. (2020). Structure of Inhibitor-Bound
Mammalian Complex I. Nat. Commun..

[89] Zhao P.-L., Wang L., Zhu X.-L., Huang X., Zhan C.-G., Wu J.-W., Yang G.-F. (2010). Subnanomolar
Inhibitor of Cytochrome
Bc1 Complex Designed by Optimizing Interaction with Conformationally
Flexible Residues. J. Am. Chem. Soc..

[90] Hao G.-F., Wang F., Li H., Zhu X.-L., Yang W.-C., Huang L.-S., Wu J.-W., Berry E. A., Yang G.-F. (2012). Computational
Discovery of Picomolar Qo Site Inhibitors of Cytochrome Bc1 Complex. J. Am. Chem. Soc..

[91] Wieferig J.-P., Kühlbrandt W. (2023). Analysis of
the Conformational Heterogeneity of the
Rieske Iron–Sulfur Protein in Complex III2 by Cryo-EM. IUCr J..

[92] Huang L., Cobessi D., Tung E. Y., Berry E. A. (2005). Binding
of the Respiratory
Chain Inhibitor Antimycin to the Mitochondrial Bc1 Complex: A New
Crystal Structure Reveals an Altered Intramolecular Hydrogen-Bonding
Pattern. J. Mol. Biol.

[93] ASPA - RISK-HUNT3R Alternative Safety Profiling Algorithm. https://www.risk-hunt3r.eu/aspa/. (Accessed 22–07–2025).

[94] Leist, M. ; Tangianu, S. ; Affourtit, F. ; Braakhuis, H. ; Colbourne, J. ; Cöllen, E. ; Dreser, N. ; Escher, S. E. ; Gardner, I. ; Hahn, S. ; An Alternative Safety Profiling Algorithm (ASPA) to Transform next Generation Risk Assessment into a Structured and Transparent Process. ALTEX, 2025. DOI: 10.14573/altex.2509081. 41099509

[95] Arcon J. P., Turjanski A. G., Martí M. A., Forli S. (2021). Biased Docking for
Protein-Ligand Pose Prediction. Methods Mol.
Biol..

[96] Luo Q., Zhao L., Hu J., Jin H., Liu Z., Zhang L. (2017). The Scoring Bias in
Reverse Docking and the Score Normalization Strategy
to Improve Success Rate of Target Fishing. PLoS
One.

[97] Rohlenova K., Sachaphibulkij K., Stursa J., Bezawork-Geleta A., Blecha J., Endaya B., Werner L., Cerny J., Zobalova R., Goodwin J., Spacek T., Alizadeh
Pesdar E., Yan B., Nguyen M. N., Vondrusova M., Sobol M., Jezek P., Hozak P., Truksa J., Rohlena J., Dong L.-F., Neuzil J. (2017). Selective Disruption
of Respiratory Supercomplexes as a New Strategy to Suppress Her2high
Breast Cancer. Antioxid. Redox Signaling.

[98] Gowthaman R., Deeds E. J., Karanicolas J. (2013). Structural Properties of Non-Traditional
Drug Targets Present New Challenges for Virtual Screening. J. Chem. Inf. Model..

[99] Wang W., Zhou X., He W., Fan Y., Chen Y., Chen X. (2012). The Interprotein Scoring Noises in
Glide Docking Scores. Proteins: Struct., Funct.,
Bioinf..

[100] Ericksen S. S., Wu H., Zhang H., Michael L. A., Newton M. A., Hoffmann F. M., Wildman S. A. (2017). Machine Learning
Consensus Scoring Improves Performance Across Targets in Structure-Based
Virtual Screening. J. Chem. Inf Model..

[101] Vass M., Kooistra A. J., Ritschel T., Leurs R., de Esch I. J., de Graaf C. (2016). Molecular Interaction Fingerprint
Approaches for GPCR Drug Discovery. Curr. Opin.
Pharmacol..

[102] Kim H., Xia D., Yu C. A., Xia J. Z., Kachurin A. M., Zhang L., Yu L., Deisenhofer J. (1998). Inhibitor
Binding Changes Domain Mobility in the Iron-Sulfur Protein of the
Mitochondrial Bc1 Complex from Bovine Heart. Proc. Natl. Acad. Sci. U. S. A..

[103] Sun X., Zheng Y., Tian L., Miao Y., Zeng T., Jiang Y., Pei J., Ahmad B., Huang L. (2021). Metabolome
Profiling and Molecular Docking Analysis Revealed the Metabolic Differences
and Potential Pharmacological Mechanisms of the Inflorescence and
Succulent Stem of Cistanche Deserticola. RSC
Adv..

[104] Grillberger K., Cöllen E., Trivisani C. I., Blum J., Leist M., Ecker G. F. (2023). Structural Insights
into Neonicotinoids and N-Unsubstituted Metabolites on Human nAChRs
by Molecular Docking, Dynamics Simulations, and Calcium Imaging. Int. J. Mol. Sci..

[105] Ibrahim R. M., Abdel-Baki P. M., Mohamed O. G., Al-Karmalawy A. A., Tripathi A., El-Shiekh R. A. (2024). Metabolites Profiling, in-Vitro and
Molecular Docking Studies of Five Legume Seeds for Alzheimer’s
Disease. Sci. Rep..

