# Supplementary material for: Leveraging Consensus Docking Approaches for Human Mitochondrial Complexes I and III
Source: Chem Res Toxicol. 2025 Dec 30;39(1):79–94. doi: 10.1021/acs.chemrestox.5c00348 (PMC12820957; doi:10.1021/acs.chemrestox.5c00348)
Supplement: Supplementary file 2 [file tx5c00348_si_002.pdf]

Supporting Information

Leveraging Consensus Docking Approaches for Human Mitochondrial Complexes I and III

Karin Grillberger †, Viktoria Magel ⊥, Marcel Leist ⊥, Gerhard F. Ecker †*

† Department of Pharmaceutical Sciences, University of Vienna, Josef-Holaubek-Platz 2, 1090 Vienna, Austria.

⊥ In vitro Toxicology and Biomedicine, Department inaugurated by the Doerenkamp-Zbinden foundation, University of Konstanz, Universitätsstraße 10, 78457 Konstanz, Germany

Supporting Information Table of Contents:

	Page
**Table S2**. Summary of performance metrics of the scoring-based classification thresholds	S2
**Figure S1.** Binding modes and redocking results at human mitochondrial CIII (cytochrome bc1 complex).	S3
**Figure S2.** Kernel density estimate (KDE) plots showing the distribution of rankings of each scoring function and consensus scores from docking into human CI (NADH:ubiquinone oxidoreductase)	S4
**Figure S3.** Kernel density estimate (KDE) plots showing the distribution of rankings of each scoring function and consensus scores from docking into human CIII (cytochrome bc_1 complex)	S5
**Figure S4**. KDE-plots (A, C, E) and confusion matrices (B, D, F) derived from the intersection points of the respective scoring functions.	S6
**Figure S5.** Interaction Profiles at mitochondrial CI. A) Stacked bar plot of CI-inhibitor's interaction counts at the CI-Qd binding site.	S7
**Figure S6.** Consensus interaction fingerprint profiles from docking into mitochondrial A) CI and B) CIII.	S8
**Figure S7.** Binding modes of piericidin A in the mitochondrial complex I (CI)-Qd binding site.	S9

Table S2. Summary of performance metrics of the scoring-based classification thresholds. MCC: Matthews Correlation Coefficient. BAC: Balanced accuracy.

Scoring Function	Target and threshold	MCC	BAC	accuracy
Glide_IFDScore	CI: −3170.75	0.786	0.577	0.786
GOLD_GoldScore_Fitness	CIII: 46.20	0.781	0.896	0.893
GOLD_PLP_Fitness	CIII: 80.52	0.647	0.824	0.786
MMGBSA dG Bind (GOLD)	CIII: −59.30	0.602	0.807	0.786

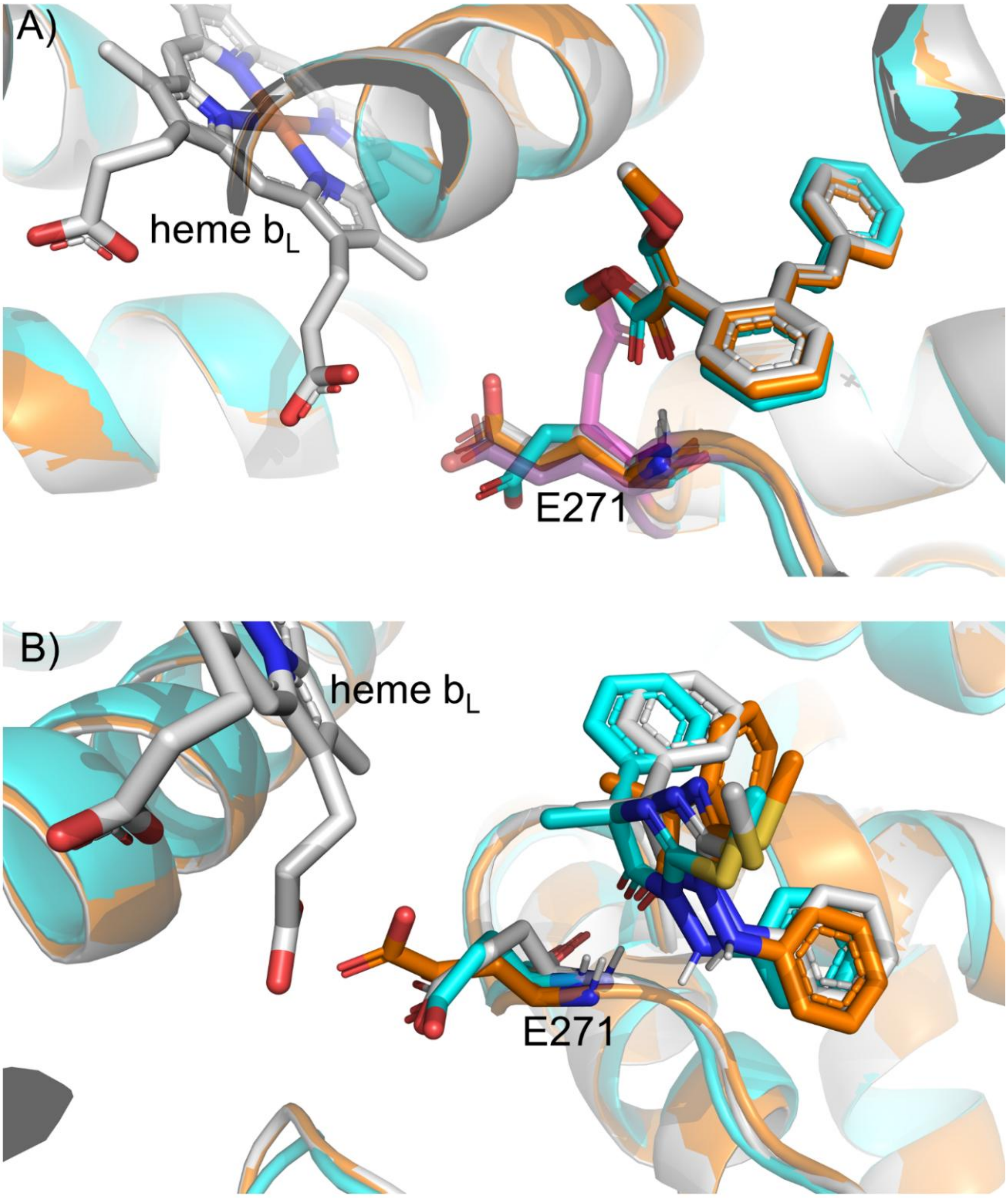

Figure S1. Binding modes and redocking results at human mitochondrial CIII (cytochrome bc_1 complex). A) Binding modes of methoxy acrylate-stilbene (MOAS) in CIII. MOAS was co-crystallized in bovine cytochrome bc1 (PDB-ID: 1sqq; cyan ribbon and carbon atoms, [1]), and the structure was superimposed with azoxystrobin docked with GOLD (orange carbon atoms and ribbon) and Maestro22-4 (grey carbon atoms and ribbon) into the human structure (PDB-ID: 5xte). Before docking into 5xte, the conformation of Glu271 was changed to adapt the apo-conformation (magenta carbon atoms of Glu271) to an inhibitor-bound conformation of the binding site, similar to in PDB-ID: 1sqq (purple carbon atoms of Glu271). The RMSD of the MOAS docking pose from GOLD and Maestro is the human structure to the co-crystallized bovine binding mode, is 1.1377 and 1.375, respectively. B) Binding modes of fenamidone in CIII. Fenamidone was co-crystallized in a bovine cytochrome bc1 complex (PDB-ID: 5klv; cyan ribbon and carbon atoms). The docked structures using GOLD (orange ribbon and carbon atoms) and Maestro22-4 (grey ribbon and carbon atoms) were superimposed and achieved a ligand RMSD of 2.069 and 1.727, respectively. Red: oxygen, blue: nitrogen, yellow: sulfur.

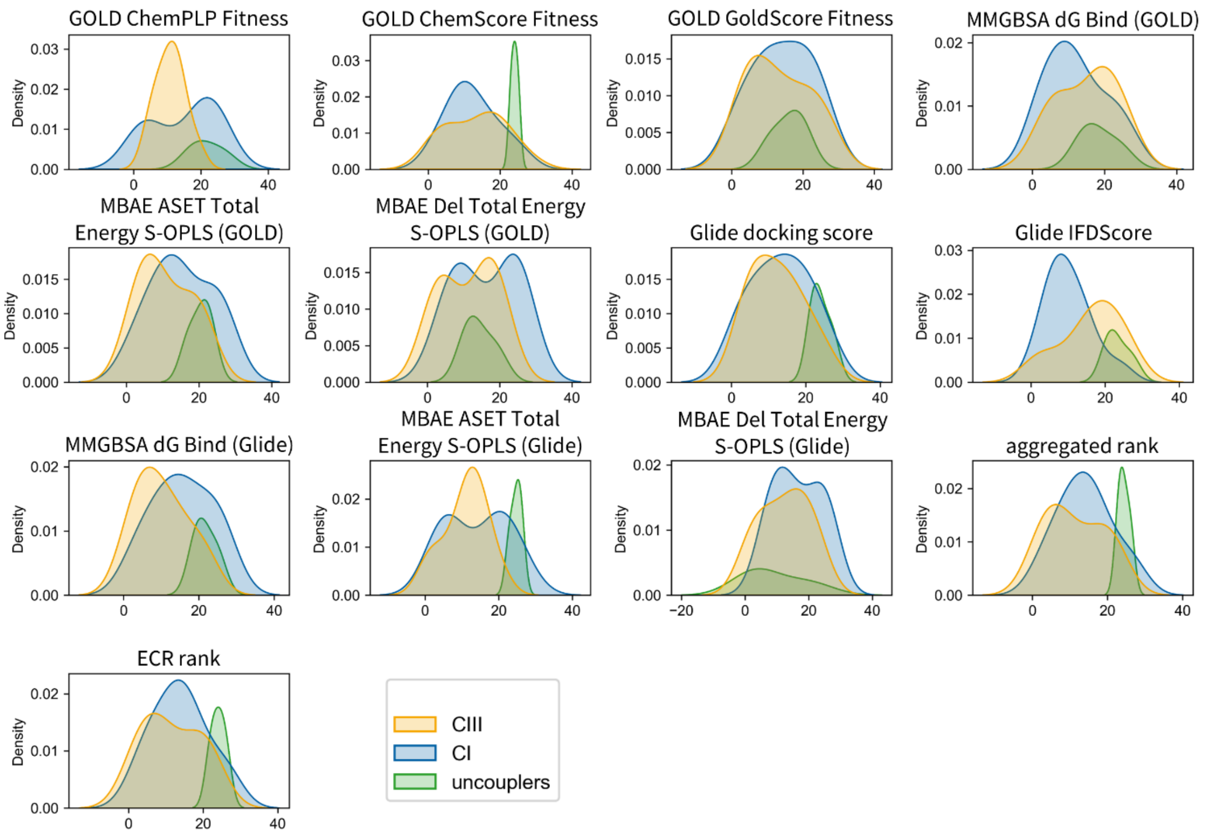

Figure S2. Kernel density estimate (KDE) plots showing the distribution of rankings of each scoring function and consensus scores from docking into human CI (NADH:ubiquinone oxidoreductase). PDB-ID 5xtd was used as structure for human CI.[2] Curves for uncoupler, CI-, and CIII-inhibitors are colored in green, blue, and yellow, respectively. Relative rankings are indicated on the x-axis and were used for this plot to compensate for different scales, units, and ranking directions of each scoring function. For scoring-based classification, a separation of the 3 density curves is desired, having the highest density of CI-inhibitors at low ranks. This worked best only for the IFDScore, whereas other scoring functions reported either major overlaps of the density curves, or incorrectly prioritized CIII-inhibitors at low ranks from docking into CI here.

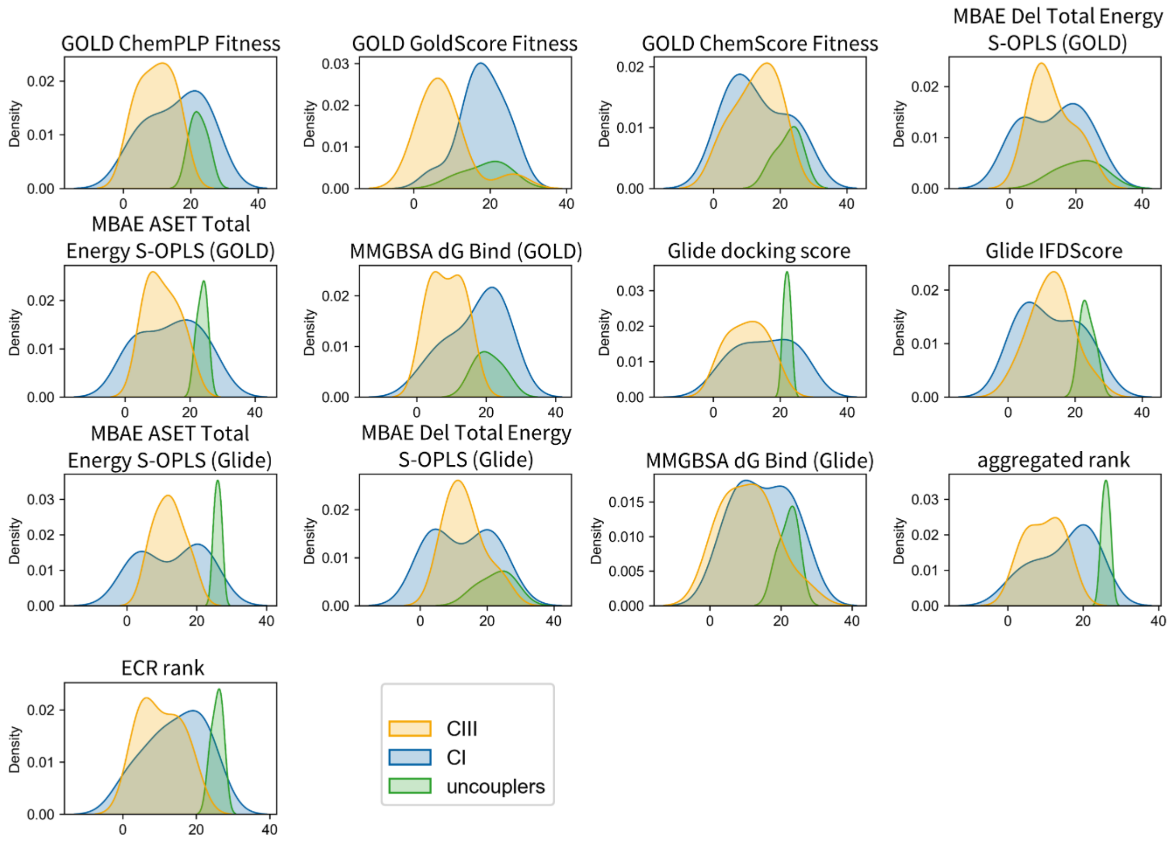

Figure S3. Kernel density estimate (KDE) plots showing the distribution of rankings of each scoring function and consensus scores from docking into human CIII (cytochrome bc_1 complex). Density curves for uncoupler, CI-, and CIII-inhibitors are colored in green, blue, and yellow, respectively. PDB-ID 5xte was used as structure for human CIII.[2] Rankings were used for this plot to compensate for different scales, units, and ranking directions of each scoring function. For scoring-based classification, a separation of the 3 density curves is desired, having the highest density of CIII-inhibitors at low ranks, which was the case for the GoldScore and ChemPLP Fitness functions from GOLD, MMGBSA dG Bind (GOLD), aggregated rank, and ECR rank.

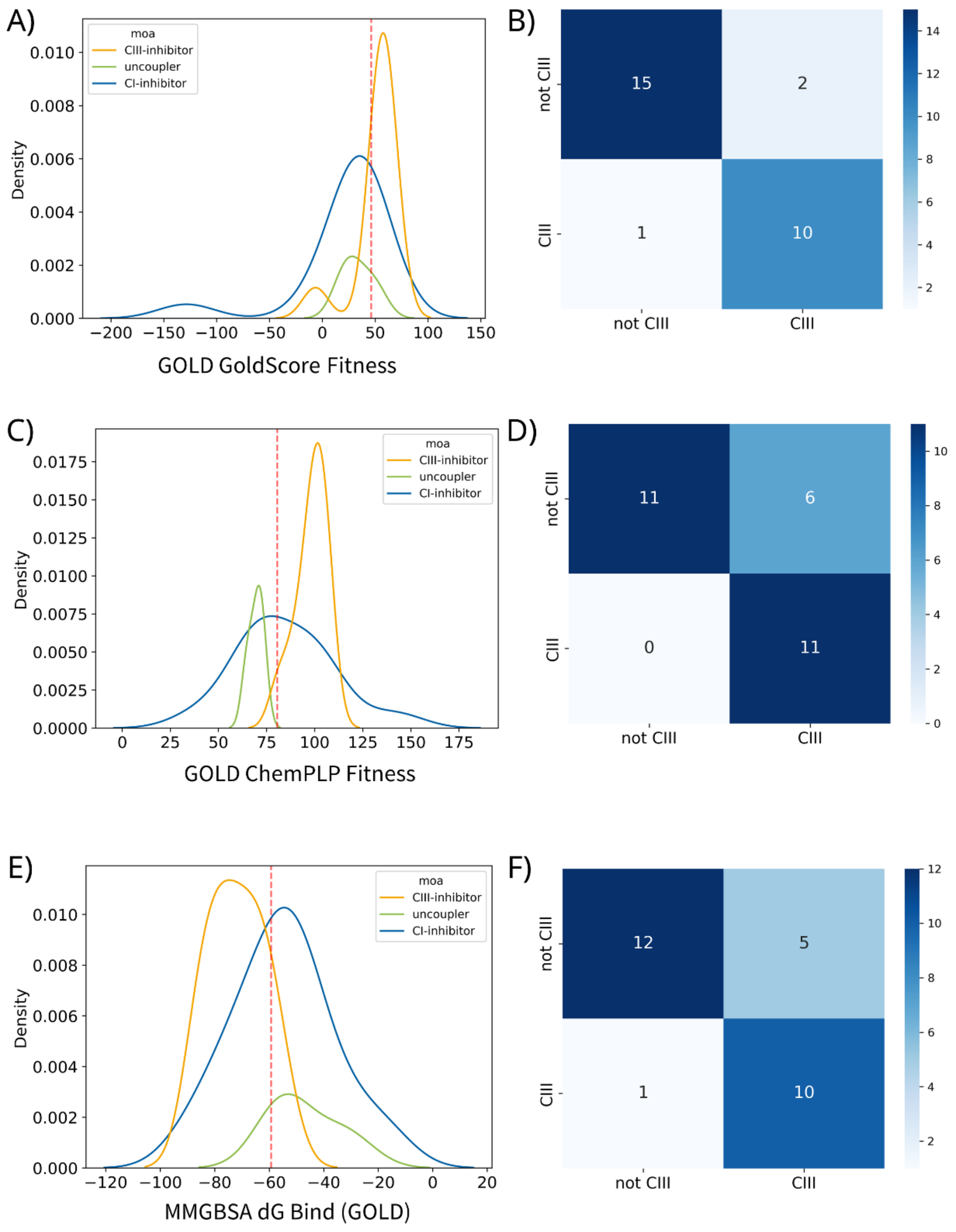

Figure S4. KDE-plots (A, C, E) and confusion matrices (B, D, F) derived from the intersection points of the respective scoring functions from docking into CIII. The KDE-curves for 28 CI- and CIII-inhibitors and uncoupling compounds are colored blue, yellow, and green, respectively. (A) The intersection point using the scoring function GOLD GoldScore is at 46.20 had the best performance according to MCC (0.781) and BAC (0.896), see also Table S2. (C) Using the ChemPLP Fitness function from GOLD reports an intersection point at a score of 80.52, which results in a confusion matrix (D) that reports an MMC value of 0.647 and BAC 0.823. (E) KDE-plot using the MMGBSA dG Bind of GOLD-generated docking poses, shows an intersection point at −59.30, that corresponds to a confusion matrix (F), MCC of 0.602, and BAC of 0.807. MCC: Matthews Correlation Coefficient. BAC: Balanced Accuracy.

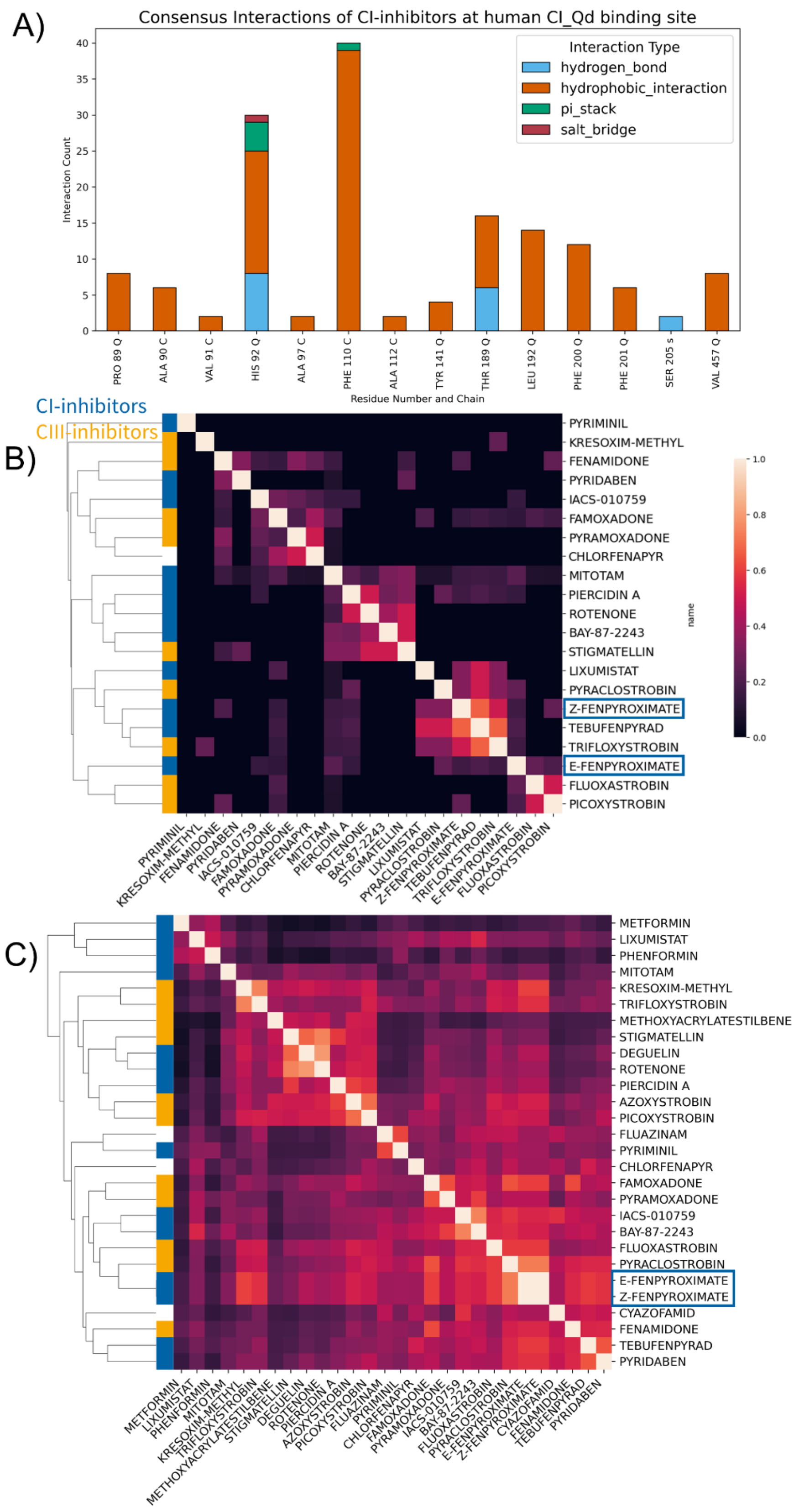

Figure S5. Interaction Profiles at mitochondrial CI. A) Stacked bar plot of CI-inhibitor's interaction counts at the CI-Qd binding site. Hydrophobic contacts are colored in orange, hydrogen bonds in blue, pi-stacking in green, and salt bridges in red. B) Hierarchically clustered heatmap of the consensus interaction fingerprint generated from consensus docking and PLIP.[3] C) Hierarchically clustered heatmap of the docked compounds and their MACCS-keys descriptors.. In the clustered heatmaps, CI- and CIII-inhibitor labels on the left

are in blue and yellow, respectively, uncoupling compounds are labelled white. The heatmaps are colored according to Tanimoto similarity, where light and orange shades indicate high similarity, and dark purple shading indicates dissimilar compounds using the respective fingerprints for comparison.

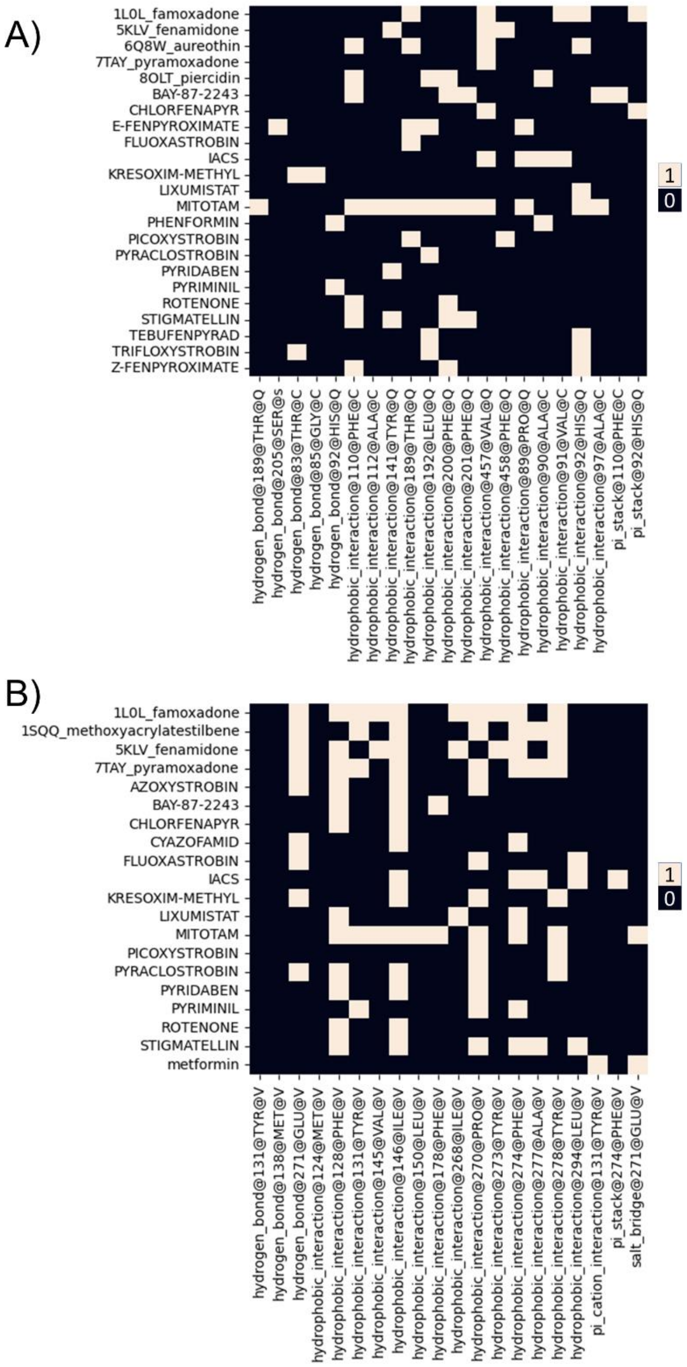

Figure S6. Consensus interaction fingerprint profiles from docking into mitochondrial A) CI and B) CIII. Rows represent ligands, and columns represent a unique combination of interaction type, amino acid number, and type and respective protein chain. If the interaction is present in the consensus interaction fingerprint, i.e., it was reported by both binding modes from the consensus docking approach, it is indicated by light coloring in the heatmap, whereas black indicates absence.

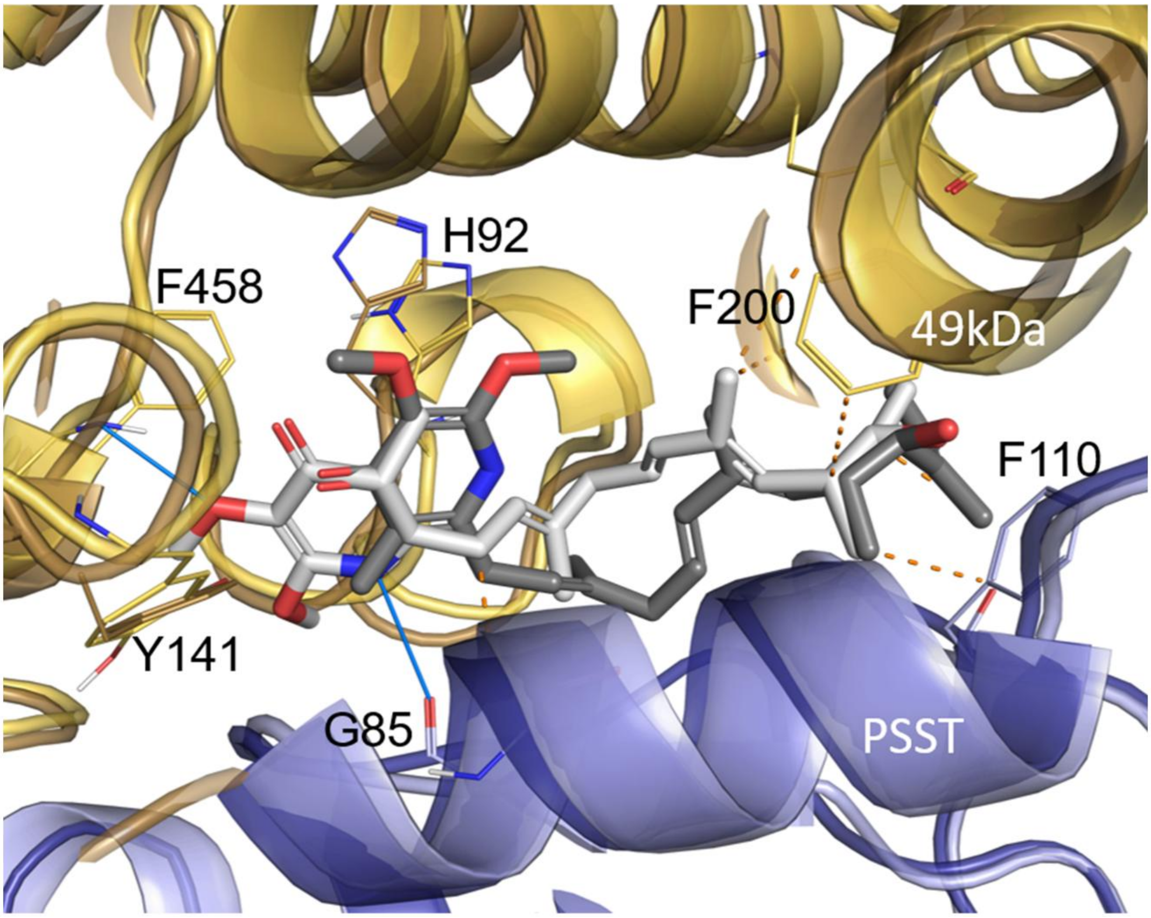

Figure S7. Binding modes and redocking results of piericidin A in the mitochondrial complex I (CI)-Qd binding site. Piericidin A was co-crystallized in the murine CI (PDB-ID 8olt[4]; dark gray carbon atoms, dark blue and yellow ribbon for the PSST- and 49kDa-subunits, respectively), and the structure was superimposed with the docked binding mode of piericidin A (light gray carbon atoms), which was generated using the IFD protocol of Schrödinger in the human structure of CI, PDB-ID: 5xtd, light blue and yellow ribbon, and binding site amino acid side chains. The RMSD of the piericidin docking pose in the human structure to the co-crystallized murine binding mode is 2.71, displaying differences in the ring conformation, but overall still acceptable alignment considering the size of the molecule and induced fit approach. Red: oxygen, blue: nitrogen.

References

(1) Esser, L.; Quinn, B.; Li, Y.-F.; Zhang, M.; Elberry, M.; Yu, L.; Yu, C.-A.; Xia, D. Crystallographic Studies of Quinol Oxidation Site Inhibitors: A Modified Classification of Inhibitors for the Cytochrome Bc1 Complex. *Journal of Molecular Biology* **2004**, *341* (1), 281–302. https://doi.org/10.1016/j.jmb.2004.05.065.

(2) Guo, R.; Zong, S.; Wu, M.; Gu, J.; Yang, M. Architecture of Human Mitochondrial Respiratory Megacomplex I2III2IV2. *Cell* **2017**, *170* (6), 1247-1257.e12. https://doi.org/10.1016/j.cell.2017.07.050.

(3) Adasme, M. F.; Bolz, S. N.; Al-Fatlawi, A.; Schroeder, M. Decomposing Compounds Enables Reconstruction of Interaction Fingerprints for Structure-Based Drug Screening. *Journal of Cheminformatics* **2022**, *14* (1). https://doi.org/10.1186/S13321-022-00592-W.

(4) Grba, D. N.; Chung, I.; Bridges, H. R.; Agip, A.-N. A.; Hirst, J. Investigation of Hydrated Channels and Proton Pathways in a High-Resolution Cryo-EM Structure of Mammalian Complex I. *Science Advances* **2023**, *9* (31), eadi1359. https://doi.org/10.1126/sciadv.adi1359.
